# Anti-Acid Drug Treatment Induces Changes in the Gut Microbiome Composition of Hemodialysis Patients

**DOI:** 10.3390/microorganisms9020286

**Published:** 2021-01-30

**Authors:** Yi-Ting Lin, Ting-Yun Lin, Szu-Chun Hung, Po-Yu Liu, Ping-Hsun Wu, Yun-Shiuan Chuang, Wei-Chun Hung, Yi-Wen Chiu, Mei-Chuan Kuo, Chun-Ying Wu

**Affiliations:** 1 Department of Family Medicine, Kaohsiung Medical University Hospital, Kaohsiung 807, Taiwan; 960254@kmuh.org.tw (Y.-T.L.); kinkipag@gmail.com (Y.-S.C.); 2 Graduate Institute of Clinical Medicine, College of Medicine, Kaohsiung Medical University, Kaohsiung 807, Taiwan; 3 Faculty of Medicine, College of Medicine, Kaohsiung Medical University, Kaohsiung 807, Taiwan; chiuyiwen@kmu.edu.tw (Y.-W.C.); mechku@kmu.edu.tw (M.-C.K.); 4 Division of Nephrology, Taipei Tzu Chi Hospital, Buddhist Tzu Chi Medical Foundation, and School of Medicine, Tzu Chi University, Hualien 970, Taiwan; water_h2o_6@hotmail.com (T.-Y.L.); szuchun.hung@gmail.com (S.-C.H.); 5 Department of Internal Medicine, National Taiwan University College of Medicine, Taipei 106, Taiwan; poyu.liu@gmail.com; 6 Division of Nephrology, Department of Internal Medicine, Kaohsiung Medical University Hospital, Kaohsiung Medical University, Kaohsiung 807, Taiwan; 7 Department of Microbiology and Immunology, Kaohsiung Medical University, Kaohsiung 807, Taiwan; wchung@kmu.edu.tw; 8 Faculty of Renal Care, College of Medicine, Kaohsiung Medical University, Kaohsiung 807, Taiwan; 9 Institute of Biomedical Informatics, Medical College, National Yang-Ming University, Taipei 112, Taiwan; dr.wu.taiwan@gmail.com; 10 Division of Translational Research, Department of Medical Research Taipei Veterans General Hospital, Taipei 112, Taiwan; 11 Department of Public Health, China Medical University, Taichung 406, Taiwan; 12 National Institute of Cancer Research, National Health Research Institutes, Miaoli 350, Taiwan

**Keywords:** microbiome, proton pump inhibitor, histamine-2 blocker, hemodialysis

## Abstract

Anti-acid drugs, proton pump inhibitor (PPI) and histamine-2 blocker (H_2_-blocker), are commonly prescribed to treat gastrointestinal disorders. These anti-acid drugs alter gut microbiota in the general population, but their effects are not known in hemodialysis patients. Hence, we investigated the microbiota composition in hemodialysis patients treated with PPIs or H_2_-blocker. Among 193 hemodialysis patients, we identified 32 H_2_-blocker users, 23 PPI users, and 138 no anti-acid drug subjects. Fecal samples were obtained to analyze the gut microbiome using 16S RNA amplicon sequencing. Differences in the microbial composition of the H_2_-blocker users, PPI users, and controls were assessed using linear discriminant analysis effect size and the random forest algorithm. The species richness or evenness (α-diversity) was similar among the three groups, whereas the inter-individual diversity (β-diversity) was different between H_2_-blocker users, PPI users, and controls. Hemodialysis patients treated with H_2_-blocker and PPIs had a higher microbial dysbiosis index than the controls, with a significant increase in the genera *Provetella 2*, *Phascolarctobacterium*, *Christensenellaceae R-7 group*, and *Eubacterium oxidoreducens group* in H_2_-blocker users, and *Streptococcus* and *Veillonella* in PPI users. In addition, compared to the H_2_-blocker users, there was a significant enrichment of the genera *Streptococcus* in PPI users, as confirmed by the random forest analysis and the confounder-adjusted regression model. In conclusion, PPIs significantly changed the gut microbiota composition in hemodialysis patients compared to H_2_-blocker users or controls. Importantly, the *Streptococcus* genus was significantly increased in PPI treatment. These findings caution against the overuse of PPIs.

## 1. Introduction

The gut microbiota is a complex ecosystem in which microbes coexist and interact with the human host. There is a bidirectional causal effect relationship in patients with chronic kidney disease (CKD) and gut microbial changes [1]. Moreover, commonly used medications are associated with distinct gut microbiota signatures [2]. Among the medications, the acid-suppressive agents, such as histamine-2 blockers (H_2_-blocker) or proton pump inhibitors (PPIs), are generally well tolerated and commonly prescribed in patients with end-stage renal disease (ESRD) [3] as the first-choice treatment for acid-related disorders [4]. However, long-term anti-acid drugs have been found to be associated with several adverse events, such as osteoporosis, fracture, hypomagnesemia, vitamin B12 deficiency, iron deficiency anemia, CKD, dementia, and pneumonia [5,6]. In addition, PPIs have been associated with an increased risk of mortality [7], major adverse cardiovascular events [8], vascular calcification [9], and hip fracture [3] in patients with kidney disease. Furthermore, the long-term reduction of gastric acid secretion by PPIs was suggested to decrease gut microbial richness, alter the composition of both gastric and intestinal microbiota, and increase oral bacteria and potentially pathogenic bacteria [10,11]. Chronic acid suppression by surgical vagotomy or chronic PPI treatment may cause hypochlorhydria and alter the intraluminal environment to promote the growth of the bacterial flora in the small intestine and increase the risk of common community-acquired enteric infections [12].

Although studies have suggested profound changes in PPI users’ gut microbiota in the general population, this has not been investigated in ESRD patients. Nonetheless, conflicting reports of gut microbial diversity change after PPI administration have been observed [10,11,13,14,15]. Therefore, our study aimed to evaluate the influence of two anti-acid drugs (H_2_-blocker and PPI) on the fecal microbiome in hemodialysis patients.

## 2. Materials and Methods

### 2.1. Study Participants

The Ethics Committee approved the study protocols of Kaohsiung Medical University Hospital (KMUHIRB-E(I)-20160095 and KMUHIRB-E(I)-20180118) and Taipei Tzu Chi Hospital (07-X01-002). Hemodialysis (HD) patients from Kaohsiung Medical University Hospital and Taipei Tzu Chi Hospital, Taiwan, were recruited between August 2017 and February 2018. Participants received regular HD three times per week, 3.5–4 h with high-flux dialyzers for each HD section. Patients with active malignancies or prescribed antibiotics within 3 months before enrollment were excluded. In total, 193 HD patients, including 32 H_2_-blocker users and 23 PPI users, were recruited and collected fecal samples for high throughput 16S ribosomal RNA gene sequencing to compare the microbiome composition between groups, H_2_-blocker users, PPI users, and controls (without H_2_-blocker or PPI) (Figure S1). All investigated anti-acid drug users (H_2_-blocker or PPI) were prescribed for at least one month.

### 2.2. Comorbidity, Laboratory, and Clinical Variables

Sociodemographic data, age, sex, dialysis vintage, arteriovenous shunt type, medical history, medications, and biochemical data were obtained for all participants from the electronic health care system records. Diabetes was defined as HbA1C 6.5% or higher or the use of oral antidiabetic agents or insulin. Hypertension was defined as 140/90 mmHg or higher or taking blood pressure-lowering drugs. The definition of cardiovascular disease included a history of myocardial infarction or was documented by coronary angiography, chronic heart failure, or a cerebrovascular accident. Blood samples were obtained after overnight fasting from patients through the arteriovenous shunt before their scheduled HD session at a single midweek dialysis session. Biochemical data included hemoglobin, albumin, high sensitivity C-reactive protein, total cholesterol, low-density lipoprotein, triglycerides, ion calcium, and phosphate from routine data within 30 days before stool sample collection. Dietary data were recorded from a modified short-form food frequency questionnaire by a licensed dietitian.

### 2.3. Fecal Sample Collection and Bacterial 16S rRNA Amplicon Sequencing

All participants provided a stool sample immediately frozen after home collection and delivered to the laboratory (Germark Biotechnology, Taichung, Taiwan) in cooler bags within 24 h via commercial transportation. DNA was extracted using a QIAamp DNA Stool Mini Kit (Qiagen, Germantown, MD, USA).

The amplicon library was constructed by amplifying the variable regions 3 and 4 (V3-V4) of the 16S rRNA gene using barcode-indexed PCR primers (341F and 805R) [16] and the 16S amplicons were sequenced (300 bp paired-end) by an Illumina MiSeq sequencer by Genomics BioScience (Taipei, Taiwan). All samples were simultaneously sequenced in the same laboratory (Germark Biotechnology, Taichung, Taiwan) to minimize batch effects. The detailed methodology of 16S rRNA amplicon sequencing and processing, as described in the supplementary method.

### 2.4. Statistical and Bioinformatics Analyses

Demographic characteristic differences between H_2_-blocker users, PPI users, or controls were determined using an ANOVA test or chi-squared test, as appropriate. A rarefaction curve was constructed to prevent methodological artifacts originating from variations in sequencing depth. The α-diversity indices were estimated to evaluate the microbiome richness indices (Chao 1), and the Kruskal–Wallis test calculated evenness (Shannon index, Simpson index, and inverse Simpson index) and the *p*-value. The β-diversity (i.e., diversity in bacterial composition between samples) was estimated by computing the Bray–Curtis distance, Jensen–Shannon divergence, or Jaccard index and visualized through a principal coordinates analysis (PCoA) to evaluate the difference in bacterial communities between anti-acid drug users and controls [17]. The sample-grouped heterogeneity of β-diversity was examined using an analysis of the similarity (permutational multivariate analysis of variance using distance matrices (PERMANOVA)) with 104 bootstrap replications. The microbial dysbiosis index (MDI) [18] was determined as the log10 of the total abundance in organisms increased in H_2_-blocker users or PPI users divided by the total abundance of organisms decreased in the controls. Co-correlation analysis was used to determine the relationships within the gut ecosystem. The sparse correlations for compositional data (SparCC) algorithm (19) is described in Supplementary Methods.

The bacterial community difference between H_2_-blocker users, PPI users, and controls by the linear discriminant analysis (LDA) of effect size (LEfSe) analysis [19], heat tree method [20], hierarchical clustering heat map, and random forest method [21]. The differential abundance analysis was also analyzed using DESeq2 methods [22]. We provide a detailed method described in the Supplementary Materials.

Considering the confounding factors, regression models were used to identify associations between the target microbiota marker and anti-acid drugs used, adjusting for age, sex, and other potential confounders. To reduce the effect of zero-inflation in the microbiome data, the matrix was normalized by dividing each feature by the respective total sample sum and transformed with log10(x + 1), where x is the normalized feature coverage as calculated in the OTUs algorithm.

Co-correlation analysis, heat tree analysis, random forest analysis, and DESeq2 were performed by MicrobiomeAnalyst [23,24]. Other statistical analyses were performed using R statistical software (version 3.5.1) and STATA statistical software (version 14).

### 2.5. Functional Annotation

Predicted functional genes were aligned with the Kyoto Encyclopedia of Genes and Genomes (KEGG) database and annotated by KEGG orthology (KO) using the R “Tax4Fun” package [25]. The KEGG metabolic modules were retrieved from the KEGG MODULE database, mapped with KOs. The Wilcoxon rank-sum test calculated the differential abundance between the two anti-acid drugs. KEGG modules were deemed present when ≥30% of the enzymes were recovered after the manual removal of overly ‘promiscuous’ enzymes (that is, present in multiple modules) before abundance calculation.

## 3. Results

### 3.1. Patient Characteristics

The baseline characteristics are reported in Table 1. The mean age was 65 ± 11.5 years in H_2_-blocker users, 68.3 ± 12.1 in PPI users, and 64.1 ± 11.0 in controls. The H_2_-blocker users were more likely to be male than PPI users or controls, with the control group having a higher blood phosphate level and single pool Kt/V than H_2_-blocker users or PPI users. The indications of PPI and H_2_-blocker used were also shown (Table 1).

### 3.2. Differences in the Gut Microbiota Profile in HD Patients

**Figure 1:**
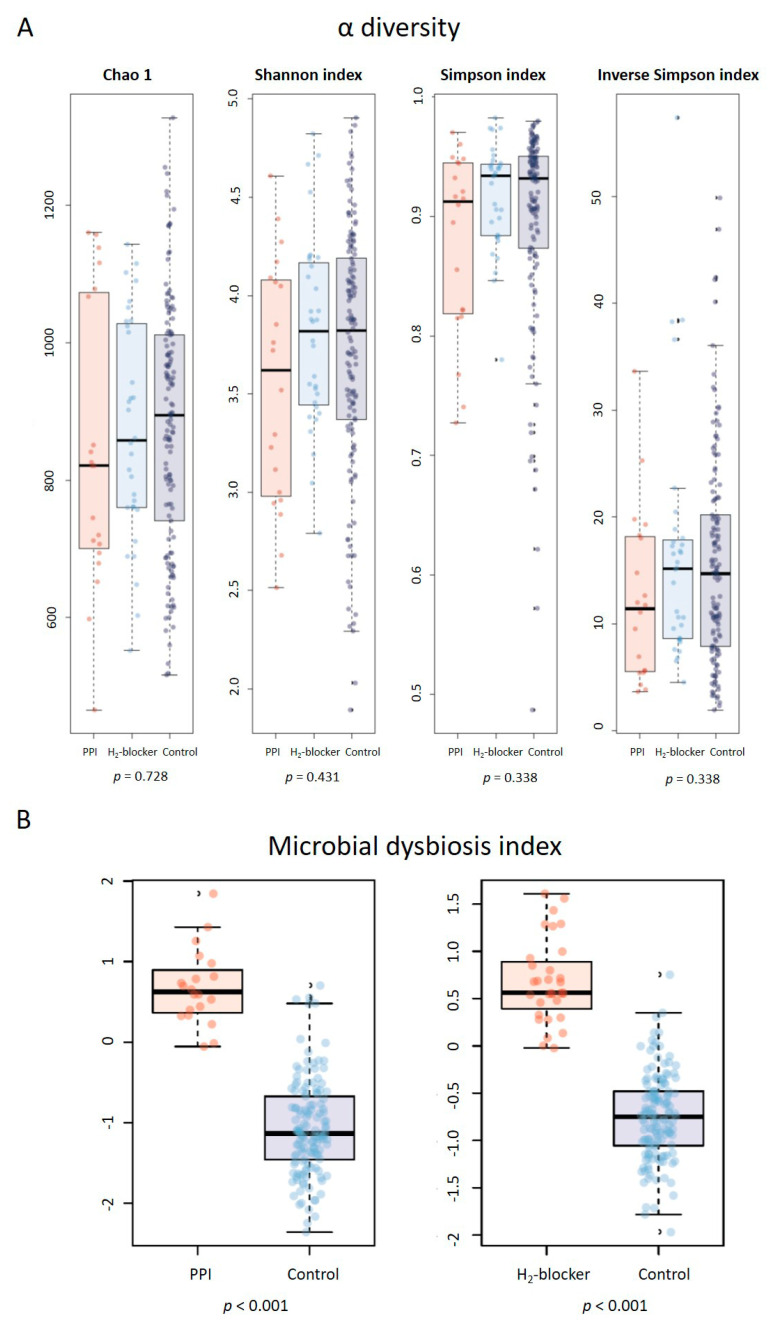
The α-diversity and microbial dysbiosis index in hemodialysis patients with proton pump inhibitor (PPI) users, H_2_-blocker users, and controls: (**A**) no difference in richness (Chao 1 index) or evenness (Shannon index, Simpson index, Inverse Simpson index); (**B**) proton pump inhibitor users or H_2_-blocker users had a higher microbial dysbiosis index compared to the controls.

**Figure 2:**
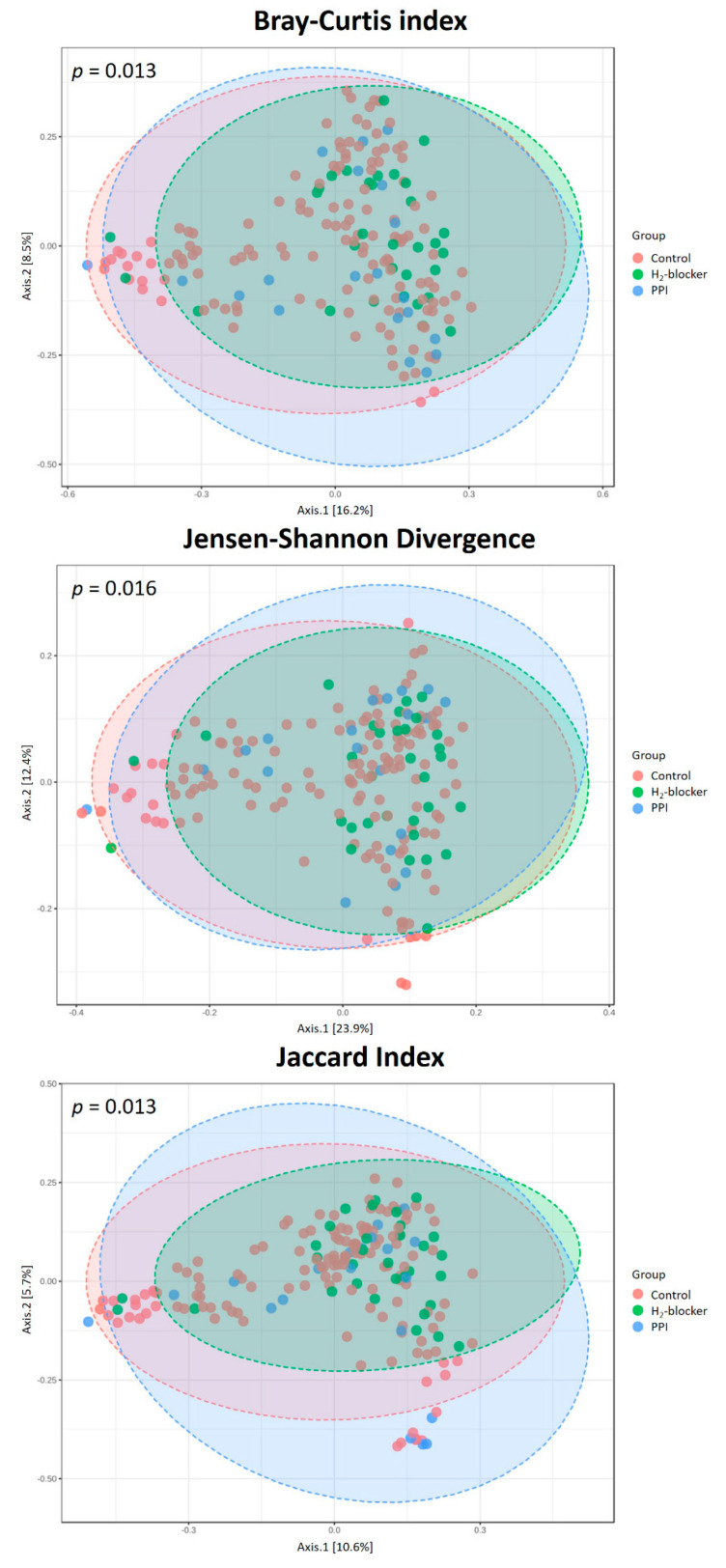
The β-diversity in hemodialysis patients with proton pump inhibitor users, H_2_-blocker users, and controls (Bray–Curtis index, Jensen–Shannon divergence, and Jaccard index). Differences in β-diversity were tested by permutational multivariate analysis of variance using distance matrices (PERMANOVA).

The rarefaction curves, which plot the OTU number as a function of the read number, showed that the three-patient groups’ contours almost overlapped, suggesting no difference in the degree of bacterial species richness (Figure S2). In addition, there was no significant difference between the groups in the relative abundance proportion (Figure S3) and α diversity (Figure 1A). However, HD patients taking H_2_-blocker or PPI had a higher MDI than controls (Figure 1B), with a significant difference in their microbiota composition (Figure 2). Similar findings were found in a subgroup analysis stratified by diabetes or not. Anti-acid users remained present at a higher MDI than the controls in both diabetic and non-diabetic patients (Figure S4).

### 3.3. Co-Occurrence Pattern Analysis of the Intestinal Ecosystems of HD Patients Treated with H_2_-Blocker, PPI and Control

Core microbiome analysis was performed at the genus level using MicrobiomeAnalyst and SparCC to calculate the Spearman correlation coefficient with the corresponding *p*-value between every two taxa (Figure S5A). The core microbiome comprised 13 genera in H2-blocker users, 11 genera in PPI users, and 12 genera in controls, with *Bacteroides*, which belong to the family *Bacteroidaceae*, being the most dominant genus (Figure S5B), followed by the core taxa belonging to *Parabacteroides* and *Lachnoclostridium*, whereas a unique core taxon, *Fusobacterium*, linked with hub taxa in the PPI network, was absent from the H_2_-blocker network.

### 3.4. Specific Microbial Taxa Are Associated with H_2_-Blocker and PPI Use

**Figure 3:**
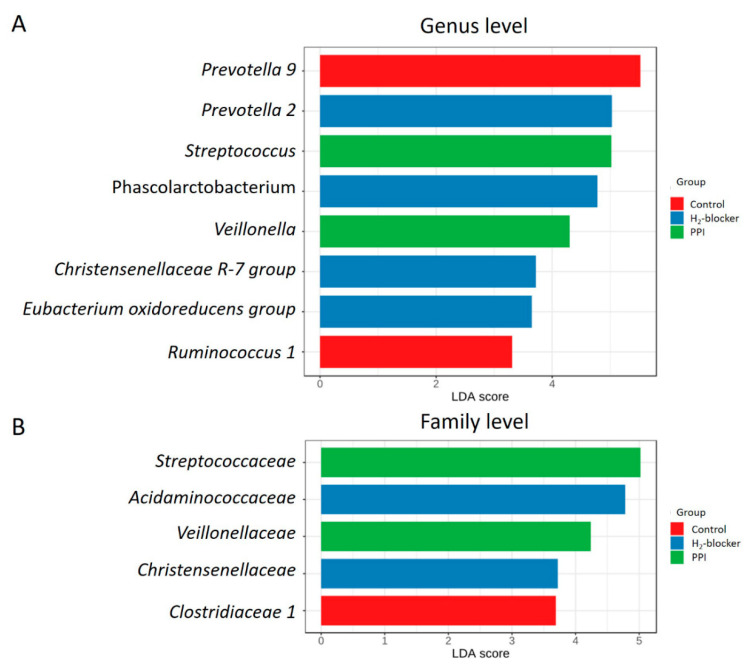
Linear discriminative analysis (LDA) effect size (LEfSe) analysis between H_2_-blocker users (blue), proton pump inhibitor users (green) and controls (red) at the (**A**) genus level and (**B**) family level.

**Figure 4:**
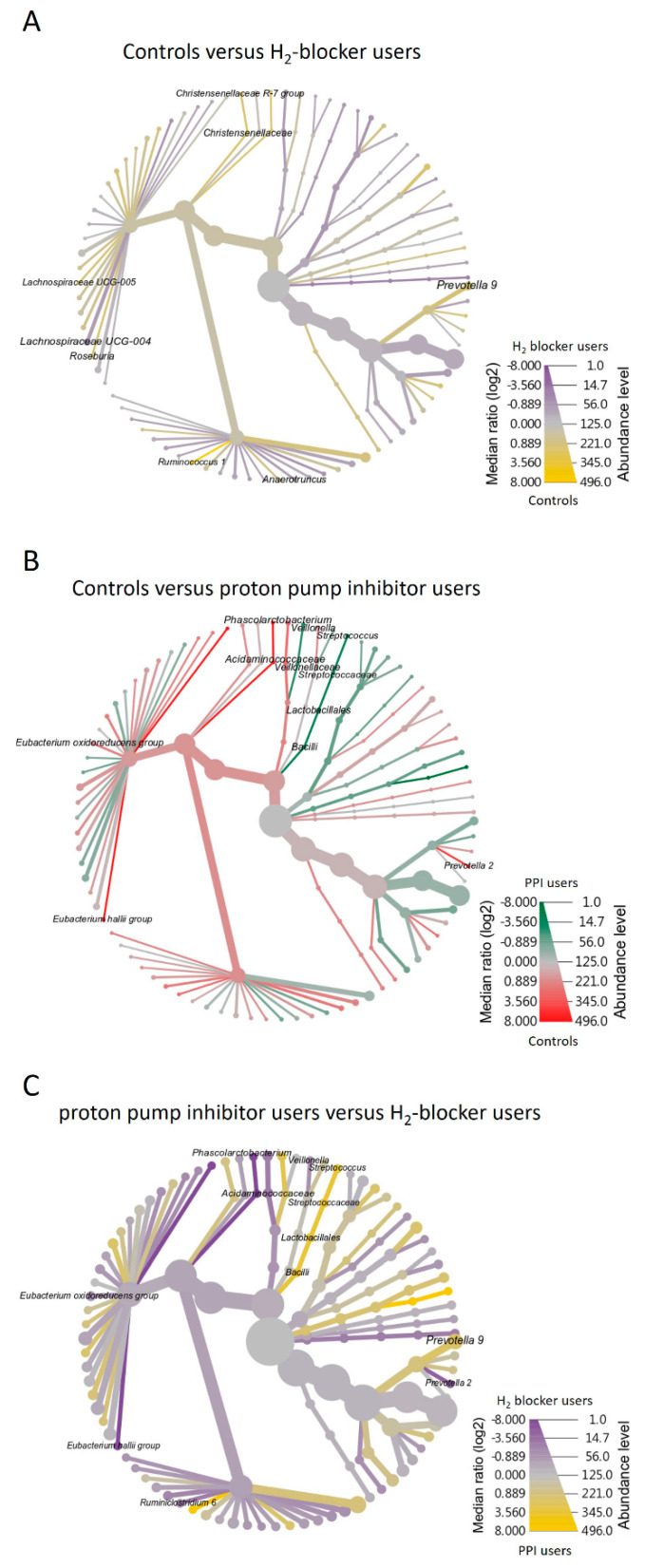
Heat tree visualization of taxonomic differences. A heat tree illustrates the taxonomic differences between H_2_-blocker users, proton pump inhibitor users, and controls. The color gradient and the size of the node, edge, and label are based on the log2 ratio of median abundance: (**A**) controls versus H_2_-blocker users; (**B**) controls versus proton pump inhibitor users; (**C**) H_2_-blocker users versus proton pump inhibitor users.

The heat map clustering analysis identified the microbial taxa that varied significantly between H_2_-blocker users and PPI users (Figure S6). The genera *Prevotella 2*, *Phascolarctobacterium*, *Christensenellaceae R-7 group*, and *Eubacterium oxidoreducens* group were enriched in H_2_-blocker users, while *Streptococcus* and *Veillonella* were enriched in PPI users and *Prevotella 9* and *Ruminococcus 1* in the controls (Figure 3A). The family *Acidaminococcaceae* and *Christensenellaceae* were enriched in the H_2_-blocker group, *Streptococcaceae* and *Veillonellaceae* in PPI users, and *Clostridiaceae 1* in controls (Figure 3B). The heat tree method revealed that compared to the controls or H_2_-blocker users, the most abundant taxa among PPI users were class *Bacilli*, order *Lactobacillales*, family *Streptoccaceae*, genus *Streptococcus*, and species *Streptococcus salivarius* (Figure 4).

**Figure 5:**
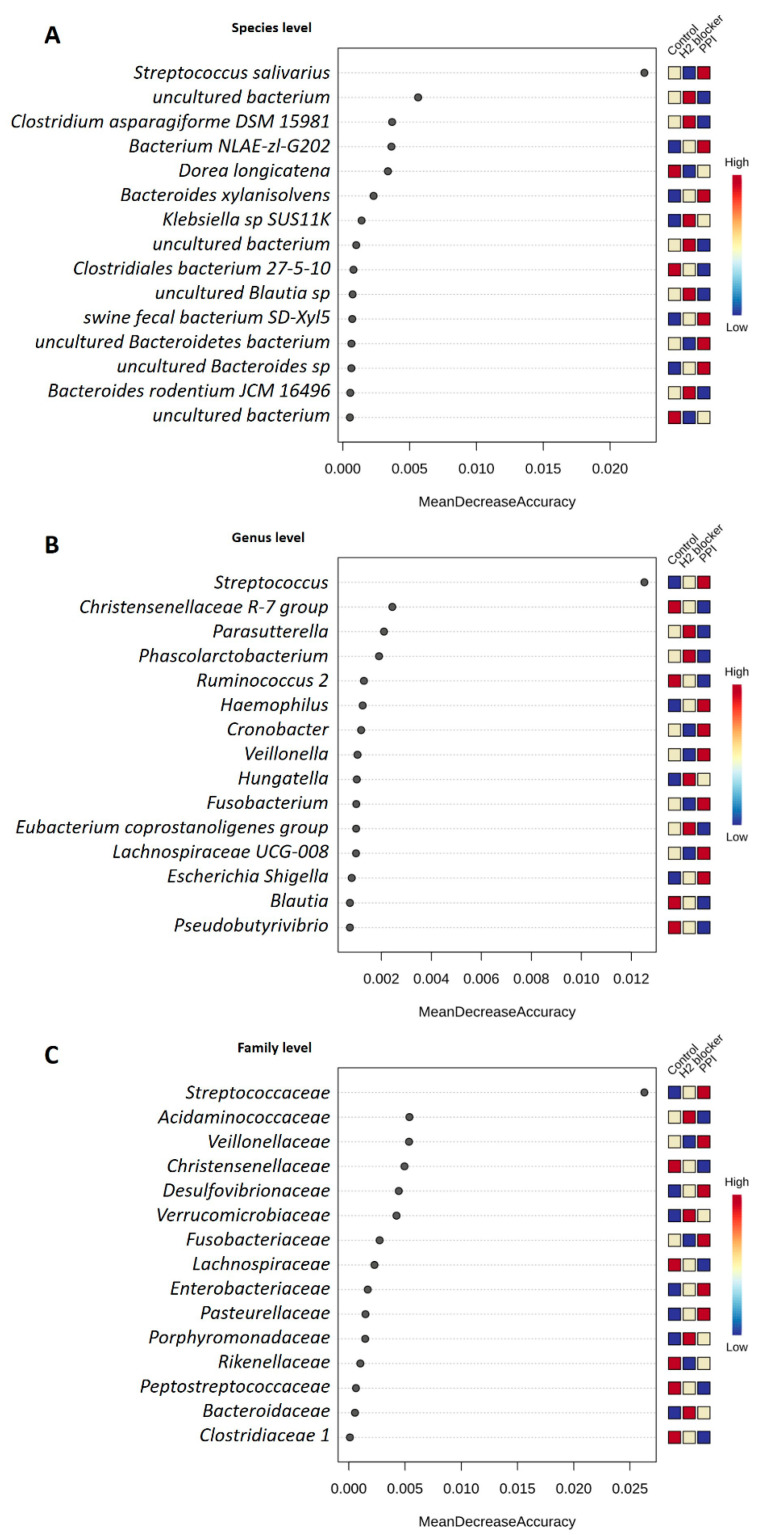
Determination of bacteria-specificity for discrimination across H_2_-blocker users, proton pump inhibitor users, and controls in hemodialysis patients. The anti-acid drugs discriminatory taxa were determined by applying random forest analysis using the (**A**) species-levels abundance; (**B**) genus-level abundance; and (**C**) family-level abundance.

Using all microbiome taxonomy from 193 samples, the machine learning random forest algorithm enabled the prediction of H_2_-blocker users, PPI users, and controls clusters with 72.6% prediction accuracy (the out-of-bag error is 0.274) in HD patients. The top-ranked bacterial taxa to discriminate between the groups were species *S. salivarius*, genus *Streptococcus*, and family *Streptococcaceae* (Figure 5). Regarding the random forest model predicted specific taxa, there was increased *S. salivarius* species, genus *Streptococcus*, and family *Streptococcaceae* in PPI users compared to H_2_-blocker users or controls. Other specific top difference taxa included less genus *Phascolarctobacterium* and family *Acidaminococcaceae* in PPI users, and more genus *Parasutterella* in H_2_-blocker users (Figure S7).

Considering confounders may influence the microbiome difference, so a multivariate-adjusted regression model was performed, showing that PPI users had higher 16S RNA levels of *Bacilli*, *Lactobacillales*, *Streptococcaceae*, and *Streptococcus* than the controls (Table 2), which remained after adjusting for covariates (age, sex, blood phosphate level, and single pool Kt/V level) in the logistic regression models.

### 3.5. Comparison of the Microbiome Differences between H2-Blocker Users and PPI Users

**Figure 6:**
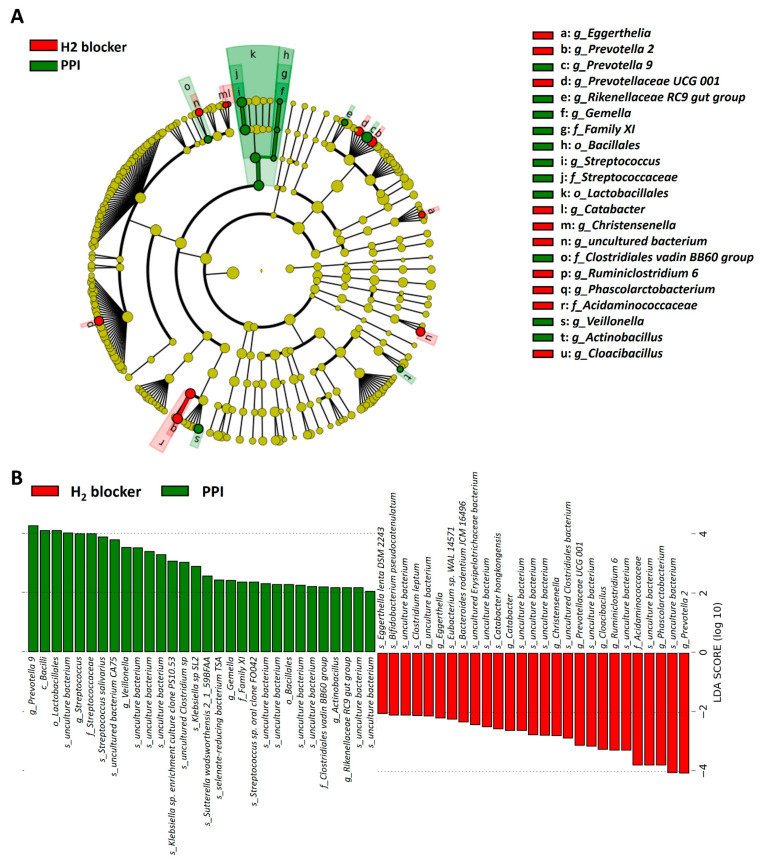
Taxonomic differences were detected between the proton pump inhibitor users and H_2_-blocker users: (**A**) cladogram showing differentially abundant taxonomic clades with an LDA score > 4.0 among PPI users and H_2_-blocker users; (**B**) linear discriminative analysis (LDA) effect size (LEfSe) analysis between proton pump inhibitor users (green) and H_2_-blocker users (red).

Microbiome differences may be related to the anti-acid effect or individual drug effect, so the differences were compared between treatment groups. The cladogram represents these differences at various phylogenic levels starting from the phylum level at the center to subphylum levels toward the periphery (Figure 6A). LDA identified an enriched relative abundance of order *Lactobacillales*, family *Streptococcaceae*, genus *Streptococcus*, and genus *Prevotella 9* in PPI users and genus *Prevotella 2* in H_2_-blocker users (Figure 6B). To demonstrate the specific microbial features associated with exposure to the different anti-acid drugs, a single microbiome taxa (genera, families, and orders) comparison was performed (Figures S8–S10). The random forest models to predict the taxonomy classification between two anti-acid drugs demonstrated similar findings (Figure S11). The abundance of the top taxa in the random forest algorithm confirmed that PPI users had higher amounts of species *S. salivarius*, genera *Streptococcus*, *Prevotella 9*, *Veillonella*, and family *Streptococcaceae* than H_2_-blocker users. In contrast, PPI users had lower amounts of species *Clostridium leptum*, *Bacteroides rodentium JCM 16496*, genera *Ruminiclostridium 6*, *Phascolarctobacterium*, and family *Acidaminococcaceae* than the H_2_-blocker users (Figure S12). The negative binomial generalized linear models (DESeq2 method) and a classical univariate method confirmed that PPI users had higher amounts of *Streptococcus* and *Veillonella* and lower amounts of *Phascolarctobacterium*, *Prevotella 2*, and *Ruminiclostridium 6* (Table S1).

### 3.6. Oral Bacterial Translocation in Anti-Acid Users

The 16S RNA amplicon sequencing was assessed against the Human Oral Microbiome Database to confirm the bacterial translocation of oral microbiota in anti-acid drug treatment, showing a different β-diversity (Bray–Curtis index, Jensen–Shannon divergence, and Jaccard index) between the three groups (Figure S13). The heat tree demonstrated an increased abundance of *Streptococcus* in PPI users than controls or H_2_-blocker users (Figure S14), specifically, *S. vestibularis* and *S. parasanguinis* clade 411 (Figure S15).

### 3.7. Functional Characterization of the Microbiome of H_2_-Blocker or PPI Users Compared to Controls

Further analysis of the KEGG modules revealed an alteration in the gut microbiota pathways in response to the H_2_-blocker or PPI use. In H_2_-blocker users, most of the mapped genes with KEGG module prediction were involved in carbohydrate metabolism (glyoxylate cycle, methylaspartate cycle), glycan metabolism (*N*-glycan precursor biosynthesis), and the metabolism of cofactors and vitamins (tetrahydrofolate biosynthesis) (Figure S16). In PPI users, the enriched predicted KEGG modules were involved in amino acid metabolism (serine biosynthesis, glutathione biosynthesis, γ-aminobutyric acid biosynthesis (GABA)), carbohydrate metabolism (ascorbate degradation), energy metabolism (nicotinamide-adenine dinucleotide phosphate (NADPH), cytochrome c oxidase, cytochrome aa3-600 menaquinol oxidase, photosystem 1, nitrogen metabolism), metabolism of cofactors and vitamins (heme biosynthesis), pathogenicity and toxins, drug resistance, and the biosynthesis of other secondary metabolites (pentalenolactone biosynthesis) (Figure S17).

## 4. Discussion

This study demonstrated that H_2_-blocker or PPI use is associated with an altered gut microbiota composition, increased MDI, and a distinct β diversity analysis compared to non-users. The microbial communities of HD patients contained higher amounts of *Bacteroidetes* and lowered Firmicutes levels, similar to CKD rat microbial communities [26]. Co-occurrence analysis revealed no significant difference in keystone taxa *Bacteroides* between H_2_-blocker users or PPI users, but there was a difference in the gut microbiota composition. Compared to controls, the genera *Provetella 2* was enriched in H_2_-blocker users and *Streptococcus* and *Veillonella* in PPI users. Furthermore, PPI users had abundant class *Bacilli* taxa, order *Lactobacillales*, family *Streptoccaceae*, genus *Streptococcus*, and species *S. salivarius*. The random forest algorithm also confirmed family *Streptoccaceae*, genus *Streptococcus*, and species *S. salivarius* as the top taxa to discriminate PPI users. Therefore, in comparison to the controls, PPI use is associated with increases in the order *Lactobacillales*, particularly the family *Streptococcaceae* and genus *Streptococcus* after adjusting for confounders.

The α-diversity analysis revealed no significant differences between H_2_-blocker users, PPI users, and controls, similar to the results reported by Clooney et al. [13], Freedberg et al. [14], and Takagi et al. [15], but not in line with Jackson et al. [11], Imhann et al. [10], and a systematic review [27]. Our HD patients had been exposed to H_2_-blocker or PPI for at least one month, similar to the drug exposure time in Freedberg et al. [14], but less than the median of 1.5 years in Jackson et al. [11]. Furthermore, the sample size was larger in Jackson et al. [11] and Imhann et al. [10]; thus, this discrepancy in results may be related to sample size and PPI treatment duration.

The distinct microbial composition in H_2_-blocker users, PPI users, and controls was demonstrated, showing that, like in previous reports [10,14,15], PPI use was associated with an increased abundance of the genera *Streptococcus* and *Veillonella*. A meta-analysis found that PPI induced a shift in the Gram-positive bacteria *Streptococcus* and *Enterococcus* [28]. As previously reported, there was also an increased abundance of *Streptococcaceae* in PPI users at the bacterial family level [11,14]. Other PPI-associated taxa reported from a systematic review [27], such as the order *Bacillales* (e.g., *Staphylococcaceae*) and *Actinomycetales* (e.g., *Actinomycetaceae*, *Micrococcaceae*), the family *Enterobacteriaceae*, *Pasteurellaceae*, *Enterococcaceae*, and *Lactobacillaceae* were not significantly different between the groups in our study; however, the role of these bacterial taxa in HD patients should be further investigated.

Several potential mechanisms may explain the change in the proximal intestinal pH that alters the gut microbiota. First, the increase in gastric pH due to anti-acid therapy may increase bacterial migration from the oral cavity to the intestinal lumen through decreased gastric acid-related bacterial killing [29], as observed by the increased oral microbiome in the fecal microbiota of PPI users, including genus *Rothia* and *Streptococcus* spp. [10,11]. In our study, members of the genus *Streptococcus*, commensals of the human oral cavity, nasopharynx, and esophagus [30], in particular, *S. salivarius*, were observed in PPI users compared to H_2_-blocker users or controls. A different β diversity and increased *Streptococcus* taxa were found using the Human Oral Microbiome Database as a reference, such as *S. vestibularis* and *S. parasanguinis clade 411*. Taken together, these findings show that bacteria present in the human oral cavity increased in the intestine, implying that bacterial translocation may have occurred. PPIs reduce gastric acidity; hence the barrier function becomes weakened, potentially accounting for the increased *Streptococcus* in our study.

Second, the diminished gastric acid secretion and small intestinal dysmotility cause the small intestinal bacterial overgrowth of microaerophilic microorganisms such as *Streptococcus*, *Staphylococcus*, *Escherichia*, and *Klebsiella* and anaerobic bacteria such as *Bacteroides*, *Lactobacillus*, *Veillonella*, and *Clostridium* [31], similar to that observed in the PPI group in our study.

Third, PPIs induce hormonal changes, including hypergastrinemia and hyperparathyroidism, which can alter the gastrointestinal bacterial milieu [32]. They can modify the luminal contents, interfering with nutrient absorption, and changing the amount or location of bacterial food substrates [33]. These potential mechanisms may explain the microbiota differences in anti-acid users compared to non-users.

The functional prediction of the microbiome between H_2_-blocker or PPI users compared to controls demonstrated several essential pathways. PPI was associated with a lower function of GABA biosynthesis compared to controls. As we know, the environmental decreasing pH is fundamental stress for cell growth during GABA production. The proton pump is also included in the acid-resistance system of lactic acid bacteria [34]. GABA biosynthesis is archived through the decarboxylation of glutamate in the cytoplasm, of which this process needs to consume intracellular protons. Therefore, PPI’s effect on GABA biosynthesis may link bacteria’s intracellular pH value [35]. In addition, PPIs could markedly reduce the proportion of vitamin C in its biologically active antioxidant form of ascorbic acid [36] because of a marked and sustained rise in intragastric pH. We also found a difference in ascorbate degradation between PPI users and controls in our study. Furthermore, PPI was associated with a lower function of heme biosynthesis compared to controls. Studies reported that the PPI-mediated reduction of gastric acid causes a reduction in the absorption of dietary iron [37,38].

This study has several limitations. First, cross-sectional studies only evaluated microbiota at a single time point, so it is impossible to capture the complex dynamics of the microbial ecosystems overtime or the microbiome alternation after the initiation of anti-acid drugs. Second, residual confounding cannot be fully excluded and statistical correlations between PPI or H_2_-blocker treatment and gut microbiota profiles do not implicate a causal relationship. Thus, studies comparing microbiota composition between anti-acid drugs naïve treatment are needed to elucidate the causal inference. Third, the sequencing of the 16S rRNA gene is limited in the analysis at the genera level. Metagenomic shotgun sequencing would permit the strain and more accurate functional analysis. Finally, the study was performed in Asian HD patients whose diet may differ from other populations, so the results should be interpreted cautiously.

Gut microbes have key roles in metabolic, nutritional, and physiological processes in the human body [39]. Changes in this microbial equilibrium, that is, dysbiosis, can promote many intestinal and extra-intestinal diseases [40,41]. In patients with kidney disease, the dysbiotic gut microbiome produced various uremic toxins and inflammation contributing to the complications [42,43]. Thus, therapeutic interventions to restore intestinal dysbiosis was recognized as a potential therapeutic target in patients with kidney disease [42,44].

Although anti-acid drugs (H_2_-blockers or PPIs) induce changes in the gut microbiota composition with unknown health-related consequences in HD patients, further consideration is the potential for the gastrointestinal tract to become a reservoir for pathogens. A significantly increased risk of community-acquired pneumonia has been observed with PPIs [45], specifically for *Streptococcus*-derived pneumonia [46]. In addition, PPI also increases sepsis risk, spontaneous bacterial peritonitis, and enteric infections [47,48]. Our study confirmed an association between PPI use and bacterial translocation using the Human Oral Microbiome Database. Given the widespread use of PPIs, healthcare providers should recognize the effects of long-term anti-acid therapy on patients’ health and avoid gut microbiota alternation-related adverse effects.

In conclusion, this study demonstrated that the use of anti-acid drugs changes the composition of gut microbiota in HD patients, with notably increased *Streptococcus* genus, *Streptococcaceae* family, and *Lactobacillales* order in PPI users.

## Figures and Tables

**Table 1:** Baseline characteristics of hemodialysis patients using the histamine-2 blocker (H_2_-blocker), proton pump inhibitor and the controls.

Baseline Characteristics	Histamine-2 Blocker Users (N = 32)	Proton Pump Inhibitor Users (N = 23)	Control Subjects (N = 138)	*p*-Value
Age (years)	65 ± 11.5	68.3 ± 12.1	64.1 ± 11.0	0.309
Male	17 (73.9%)	12 (37.5%)	77 (55.8%)	0.026
Dialysis vintage (months)	84.37 ± 52.55	98.09 ± 61.51	92.02 ± 61.73	0.778
**Cause of ESRD**				
Hypertension	1 (4.3%)	2 (6.3%)	14 (10.1%)	0.566
Diabetes mellitus	11 (47.8%)	12 (37.5%)	43 (31.2%)	0.270
Glomerulonephritis	6 (26.1%)	13 (40.6%)	56 (40.6%)	0.408
Others *	5 (21.7%)	5 (15.6%)	25 (18.1%)	0.845
**Comorbidities**				
Diabetes mellitus	13 (56.5%)	12 (37.5%)	54 (39.1%)	0.265
Hypertension	18 (78.3%)	27 (84.4%)	122 (88.4%)	0.388
Dyslipidemia	9 (39.1%)	12 (37.5%)	34 (24.6%)	0.169
**Medications**				
Anti-hypertensive drugs	17 (73.9%)	22 (68.8%)	79 (57.2%)	0.198
Diabetes treatment medications	9 (39.1%)	9 (28.1%)	39 (28.3%)	0.561
Calcium carbonate	18 (78.3%)	23 (71.9%)	120 (87.0%)	0.092
**Clinical laboratory data**				
Hemoglobin (g/dL)	10.51 ± 1.10	10.64 ± 1.09	10.7 ± 1.38	0.517
Albumin (g/dL)	3.54 ± 0.71	3.52 ± 0.52	3.55 ± 0.42	0.832
High sensitivity CRP (mg/dL)	3.4 ± 4.04	1.65 ± 4.12	2.35 ± 4.50	0.574
Total calcium (mg/dL)	9.27 ± 0.99	9.14 ± 1.10	9.24 ± 0.85	0.901
Phosphate (mg/dL)	4.63 ± 1.35	4.69 ± 1.19	5.14 ± 1.20	0.020
Single pool Kt/V	1.55 ± 0.14	1.65 ± 0.29	1.68 ± 0.28	0.046
**Dietary intake (serving/day)**				
Meat	0.86 ± 0.63	0.91 ± 0.63	0.82 ± 0.51	0.695
Vegetable	1.51 ± 1.20	1.8 ± 1.01	2.02 ± 1.09	0.083
Fruit	0.8 ± 0.90	0.84 ± 0.54	0.99 ± 0.72	0.399
Bristol stool scale	3.96 ± 1.77	4 ± 1.95	3.76 ± 1.78	0.745
**Anti-acid drugs indication**				
Peptic ulcer disease	8 (25%)	11 (47.8%)		
Gastroesophageal reflux disease	15 (46.9%)	10 (43.5%)		
Others **	9 (28.1%)	2 (8.7%)		

* Other causes of end-stage renal disease include polycystic kidney disease, tumor, systemic lupus erythematosus, gout, interstitial nephritis. ** Other indications for anti-acid drugs used: gastrointestinal bleed prophylaxis in patients on antiplatelet or anticoagulation therapy or functional dyspepsia. Abbreviation: ESRD, end-stage renal disease; CRP, C-reactive protein

**Table 2:** Distribution of the *Bacilli* class and its major subclass between and proton pump inhibitor users and controls.

Taxonomic Level	Taxon	PPI Users (*n* = 23) Reads Count, Mean ± SD	Controls (*n* = 138) Reads Count, Mean ± SD	*p*-Value, Crude	*p*-Value, Adjusted *
Class	*Bacilli*	1093.1 ± 2121.2	34.9 ± 76.9	<0.001	<0.001
Order	*Lactobacillales*	1092.5 ± 2120.5	34.6 ± 76.9	<0.001	<0.001
Family	*Streptococcaceae*	826.4 ± 2047.8	20.5 ± 36.9	<0.001	<0.001
Genus	*Streptococcus*	826.4 ± 2047.8	20.5 ± 36.9	<0.001	<0.001

SD, standard deviation. * *p*-value calculated by the logistic regression model adjusted for age, sex, blood phosphate level, and single pool Kt/V level.

## Data Availability

The data (sequenced reads) presented in this study are publicly available on National Center for Biotechnology Information’s (NCBI) Sequence Read Archive (SRA) under BioProject accession number PRJNA648014.

## Supplementary Materials

The following are available online at https://www.mdpi.com/2076-2607/9/2/286/s1, Supplementary methods, Figure S1: Enrollment of study participants; Figure S2: Rarefaction curves based on gene count in H_2_-blocker users, proton pump inhibitor users, and controls; Figure S3: The relative percentage abundance of intestinal microbiota at the phylum level between H_2_-blocker users, proton pump inhibitor users, and controls: (A) phylum, (B) class, and (C) order level; Figure S4: Subgroup analysis of α-diversity and microbial dysbiosis index in hemodialysis patients with proton pump inhibitor users, H_2_-blocker users, and controls stratified by with and without diabetes mellitus: (A) diabetic patients and (B) non-diabetic patients; Figure S5: Core microbiome analysis in H_2_-blocker users, proton pump inhibitor users, and controls; (A) SparCC correlation analysis (genus level using 100 SparCC permutations, 0.4 correlation threshold, and 0.05 *p*-value threshold); (B) relative abundance and sample prevalence of bacterial genus in H_2_-blocker users, proton pump inhibitor users, and controls; Figure S6: Hierarchical clustering heat map of bacterial genera (A) and families (B) between H_2_-blocker users, proton pump inhibitor users, and controls generated by MicrobiomeAnalyst using Euclidean distance measure and Ward clustering algorithm; Figure S7: The abundance of the top taxa in random forest algorithm and their enriched difference between H_2_-blocker users, proton pump inhibitor users, and controls: (A) species level, (B) genus level, and (C) family level; Figure S8: The relative abundance of the specific microbiome genus differentially enriched in the clinical settings after linear discriminant analysis between the H_2_-blocker and proton pump inhibitor users; Figure S9: The relative abundance of the specific microbiome family differentially enriched in the clinical settings after linear discriminant analysis; Figure S10: The relative abundance of the specific microbiome order differentially enriched in the clinical settings after linear discriminant analysis; Figure S11: Determination of bacteria-specific for discriminatory across H_2_-blocker and proton pump inhibitor users in hemodialysis patients. The anti-acid drugs discriminatory taxa were determined by applying random forest analysis using the (A) species-levels abundance, (B) genus-level abundance, and (C) family-level; Figure S12: The abundance of the top taxa in the random forest algorithm and their enriched difference between H_2_-blocker and proton pump inhibitor users: (A) species level, (B) genus level, and (C) family level; Figure S13: The β-diversity in hemodialysis patients with proton pump inhibitor users, H_2_-blocker users, and controls using Human Oral Microbiome Database as the reference database (Bray–Curtis index, Jensen–Shannon divergence, and Jaccard index). Differences in β-diversity were tested by PERMANOVA; Figure S14: Heat tree visualization of taxonomic differences based on human oral microbiome database. A heat tree illustrates the taxonomic differences between H_2_-blocker users, proton pump inhibitor users, and controls. The color gradient and the size of the node, edge, and label are based on the log2 ratio of median abundance: (A) proton pump inhibitor users versus controls and (B) proton pump inhibitor users versus H_2_-blocker users; Figure S15: The abundance of the streptococcus taxa (species level, genus level, and family level) difference between H_2_-blocker users, proton pump inhibitor users, and controls based on human oral microbiome database; Figure S16: Selected significant functional classification of the predicted metagenome content of the microbiota of H_2_-blocker users and controls by KO modules. The relative abundances of modules were compared among hemodialysis patients with and without H_2_-blocker used. Significance was considered for *p* < 0.05; Figure S17: Selected significant functional classification of the predicted metagenome content of the microbiota of proton pump inhibitor users and controls by KO modules. The relative abundances of modules were compared among hemodialysis patients with and without proton pump inhibitors used. Significance was considered for *p* < 0.05; Table S1: Summary table of significant genus difference between the H_2_-blocker and proton pump inhibitor users in negative binomial generalized linear models (DESeq2 method) and classical univariate method.
