# Supplementary material for: Anti-Acid Drug Treatment Induces Changes in the Gut Microbiome Composition of Hemodialysis Patients
Source: Microorganisms. 2021 Jan 30;9(2):286. doi: 10.3390/microorganisms9020286 (PMC7910989; doi:10.3390/microorganisms9020286)
Supplement: Supplementary file 1 [file microorganisms-09-00286-s001.pdf]

Supplementary Methods

Bacterial 16S rRNA Amplicon Sequencing and Processing

The 16S-amplicon processing pipeline was modified from the 16S Bacteria/Archaea SOP v1 of Microbiome Helper workflows [1]. Paired-end reads were merged raw reads using the Paired-End Read Merger (PEAR; version 0.9.8) [2] and filtered low-quality reads using the following thresholds of sequence length ≥400 bp and quality score of 90% bases of reads ≥20. Processing the raw sequencing reads was demultiplexed and quality filtered using the Quantitative Insight Into Microbial Ecology (QIIME; version 1.9.1) software [3].

After filtering, the sequences with 97% similarity of operational taxonomic units (OTU) from SILVA (version 123) taxonomic database [4] were clustered using the UCLUST (a centroid-based, medium to high-identity clustering) algorithm [5] with a 97% sequence identity threshold. Reads were dereplicated and singletons were discarded. The final OTU table was rarefied into minimum sequencing depth in the data set.

To evaluate the oral microbiota's bacterial translocation in anti-acid drug treatment, sequencing data were also assessed against the Human Oral Microbiome Database (HOMD) (version 14.51) [6]. OTUs were assigned a taxonomic identification based on the consensus assignment for most sequences within each OTU. In such cases, the OTU's representative sequence was also compared to the HOMD if results showed more than 97% similarity to an oral taxon.

Co-Correlation Analysis

Co-correlation analysis was evaluated by building the co-occurrence networks of the most abundant OTU shared by at least 20% of the samples and 0.2% relative abundance calculated using the Sparse Correlations for Compositional data (SparCC) algorithm [7]. The *p*-values were calculated using a bootstrap procedure with 100 random permutations and iterations for each SparCC calculation; then, the correlation matrices were computed from the resampled data matrices. Only OTUs with correlation scores greater than 0.4 and a *p*-value less than 0.05 were classified into co-abundance groups (CAGs); these coefficients were also used to assess the length of edges on a fast greedy modularity optimization algorithm to identify clusters in the network. An undirected network, weighted by SparCC correlation magnitude, was generated using bioinformatics tools in MicrobiomeAnalyst [8,9].

Linear Discriminant Analysis (LDA) of Effect Size (LEfSe) Analysis

The bacterial community difference between H_2-blocker users, PPI users, and controls by the linear discriminant analysis (LDA) of effect size (LEfSe) analysis at the OTU level with more than 0.1% relative abundances and present in >30% of samples were used to determine the most discriminatory taxa between groups. The LEfSe analysis employed the non-parametric factorial Kruskal–Wallis test or Wilcoxon rank-sum test and LDA to identify differentially abundant taxa, with taxa with an LDA score greater than 2 or less than 2 at a $p<0.05$ considered significantly enriched. LEfSe analysis was also performed to compare the bacterial communities of H_2-blocker and PPI users. Differential abundance analysis was performed based on the relative abundance levels of the genera, families, and orders after LDA selection between H_2-blocker and PPI users.

Heat Tree Method

The heat tree method was used to compare the abundance of different taxonomic levels for each pair of factors in a metadata variable. A hierarchical structure of taxonomic classifications to quantitatively (median abundance) and statistically (non-parameter Wilcoxon rank-sum test) depict taxon differences among communities was performed using the R “metacoder” package [10]. The hierarchical clustering heat map was created using the Euclidean distance measure and Ward clustering algorithm.

Random Forest Method

The random forest method [11] was used to determine a ranked list of all bacterial taxa and identify the most important predictive bacterial communities to classify H_2-blocker users, PPI users, and controls. The random forest supervised learning algorithm can rank OTUs based on their ability to discriminate among the groups considering the complex interrelationships in high dimensional data. The log-transformed read counts difference of the top selected taxa on the levels of the species, genera, and families based on the random forest algorithm between H_2-blocker users, PPI users, and controls were analyzed.

DESeq2 Method

To determine and confirm the differentially abundant genera in H_2-blocker and PPI users, the DESeq2 method was performed with an adjusted *p*-value cut off at 0.05. The DESeq2 methods initially developed for RNA-Seq count data (i.e., approximated to Poisson distribution, positive skewed) to handle the data normalization and evaluate variance or dispersion. The DESeq2 variance estimations are based on modeling the counts to a negative binomial generalized linear model to estimate dispersion and logarithmic fold changes [12].

Reference List

1. Comeau, A.M.; Douglas, G.M.; Langille, M.G. Microbiome Helper: a Custom and Streamlined Workflow for Microbiome Research. *mSystems* **2017**, *2*, doi:10.1128/mSystems.00127-16.
2. Zhang, J.; Kobert, K.; Flouri, T.; Stamatakis, A. PEAR: a fast and accurate Illumina Paired-End reAd mergeR. *Bioinformatics* **2014**, *30*, 614-620, doi:10.1093/bioinformatics/btt593.
3. Caporaso, J.G.; Kuczynski, J.; Stombaugh, J.; Bittinger, K.; Bushman, F.D.; Costello, E.K.; Fierer, N.; Pena, A.G.; Goodrich, J.K.; Gordon, J.I., et al. QIIME allows analysis of high-throughput community sequencing data. *Nat Methods* **2010**, *7*, 335-336, doi:10.1038/nmeth.f.303.
4. Yilmaz, P.; Parfrey, L.W.; Yarza, P.; Gerken, J.; Pruesse, E.; Quast, C.; Schweer, T.; Peplies, J.; Ludwig, W.; Glockner, F.O. The SILVA and "All-species Living Tree Project (LTP)" taxonomic frameworks. *Nucleic Acids Res* **2014**, *42*, D643-648, doi:10.1093/nar/gkt1209.
5. Edgar, R.C. Search and clustering orders of magnitude faster than BLAST. *Bioinformatics* **2010**, *26*, 2460-2461, doi:10.1093/bioinformatics/btq461.
6. Chen, T.; Yu, W.H.; Izard, J.; Baranova, O.V.; Lakshmanan, A.; Dewhirst, F.E. The Human Oral Microbiome Database: a web accessible resource for investigating oral microbe taxonomic and genomic information. *Database (Oxford)* **2010**, *2010*, baq013, doi:10.1093/database/baq013.
7. Friedman, J.; Alm, E.J. Inferring correlation networks from genomic survey data. *PLoS Comput Biol* **2012**, *8*, e1002687, doi:10.1371/journal.pcbi.1002687.
8. Dhariwal, A.; Chong, J.; Habib, S.; King, I.L.; Agellon, L.B.; Xia, J. MicrobiomeAnalyst: a web-based tool for comprehensive statistical, visual and meta-analysis of microbiome data. *Nucleic Acids Res* **2017**, *45*, W180-W188, doi:10.1093/nar/gkx295.
9. Chong, J.; Liu, P.; Zhou, G.; Xia, J. Using MicrobiomeAnalyst for comprehensive statistical, functional, and meta-analysis of microbiome data. *Nat Protoc* **2020**, 10.1038/s41596-019-0264-1, doi:10.1038/s41596-019-0264-1.
10. Foster, Z.S.; Sharpton, T.J.; Grunwald, N.J. Metacoder: An R package for visualization and manipulation of community taxonomic diversity data. *PLoS Comput Biol* **2017**, *13*, e1005404, doi:10.1371/journal.pcbi.1005404.
11. Svetnik, V.; Liaw, A.; Tong, C.; Culberson, J.C.; Sheridan, R.P.; Feuston, B.P. Random forest: a classification and regression tool for compound classification and QSAR modeling. *J Chem Inf Comput Sci* **2003**, *43*, 1947-

1958, doi:10.1021/ci034160g.

12. Love, M.I.; Huber, W.; Anders, S. Moderated estimation of fold change and dispersion for RNA-seq data with DESeq2. *Genome Biol* **2014**, *15*, 550, doi:10.1186/s13059-014-0550-8.

Figure S1. Enrollment of study participants.

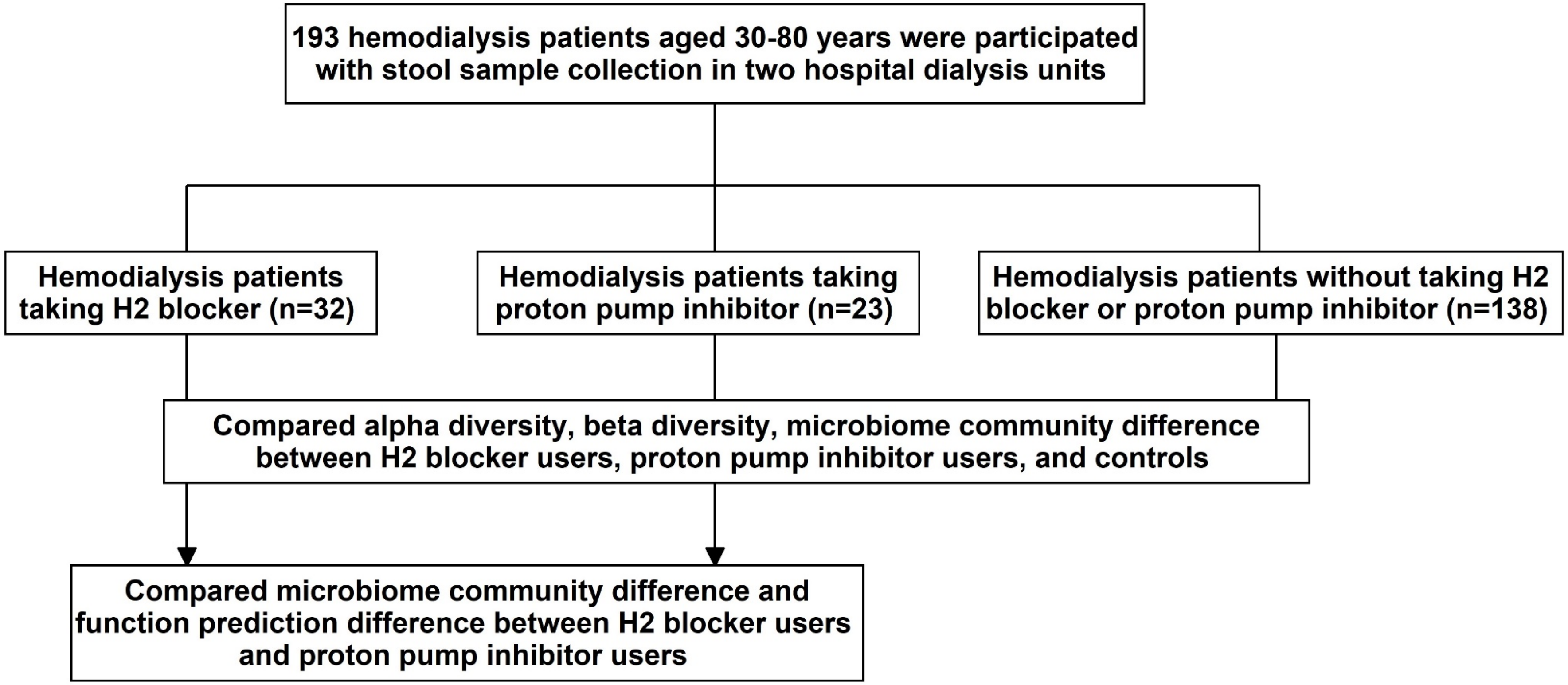

Figure S2. Rarefaction curves based on gene count in H_2-blocker users, proton pump inhibitor users, and controls.

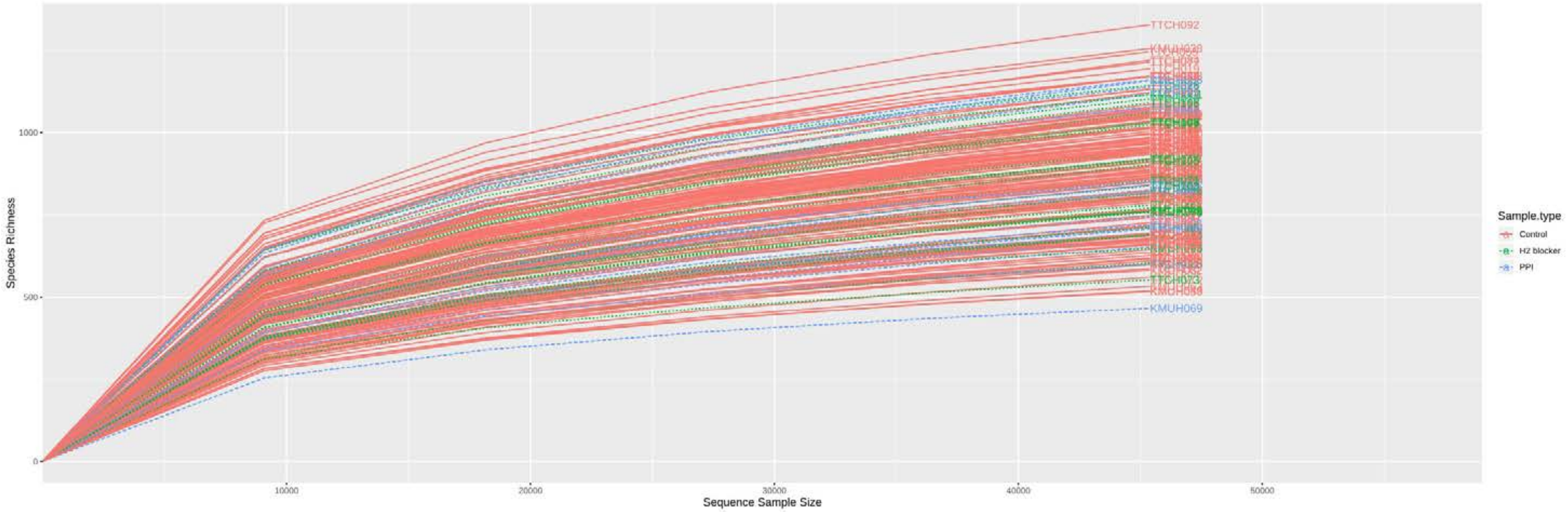

Figure S3. The relative percentage abundance of intestinal microbiota at the phylum level between H_2-blocker users, proton pump inhibitor users, and controls: (A) phylum, (B) class, and (C) order level.

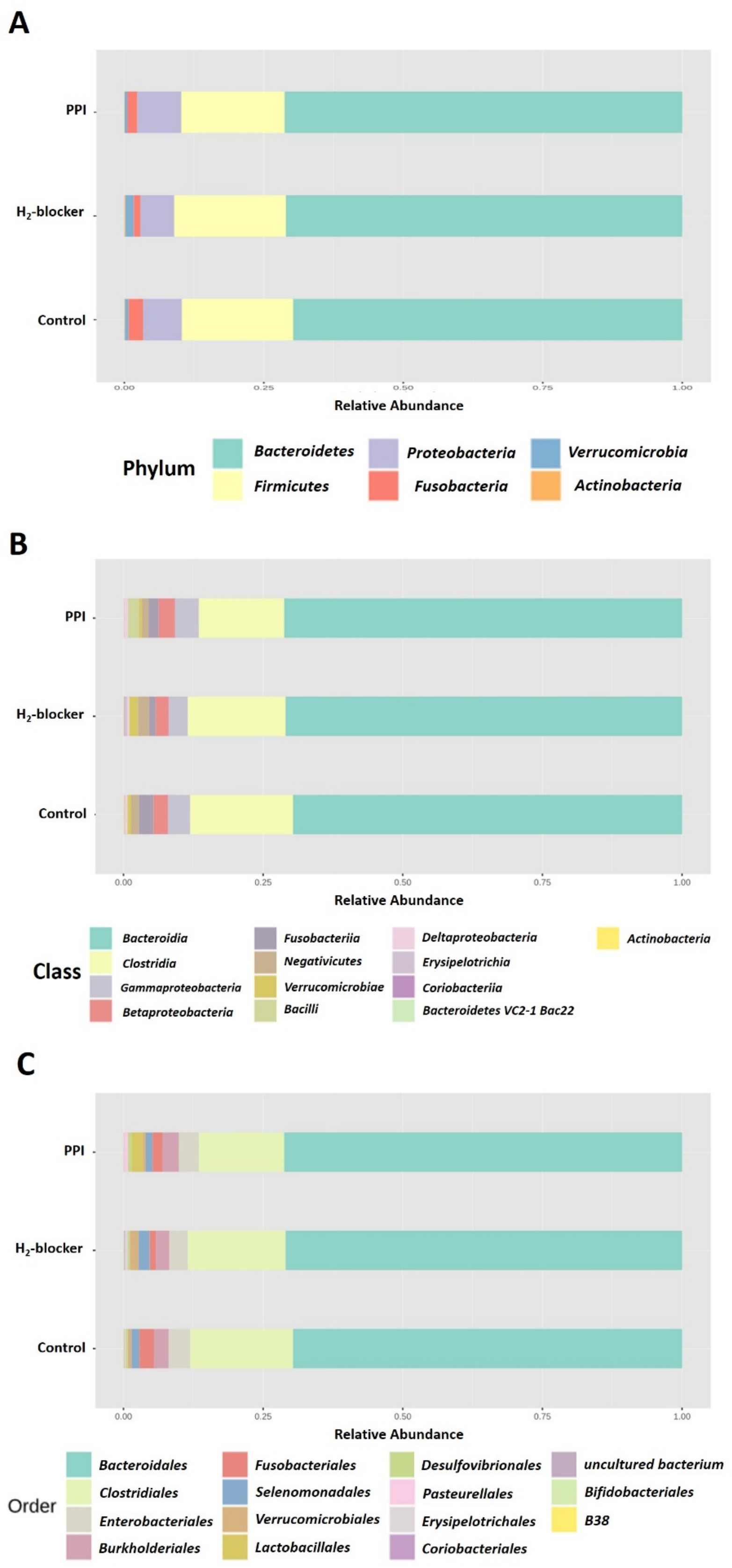

Figure S4. Subgroup analysis of α-diversity and microbial dysbiosis index in hemodialysis patients with proton pump inhibitor users, H_2-blocker users, and controls stratified by with and without diabetes mellitus: (A) diabetic patients and (B) non-diabetic patients.

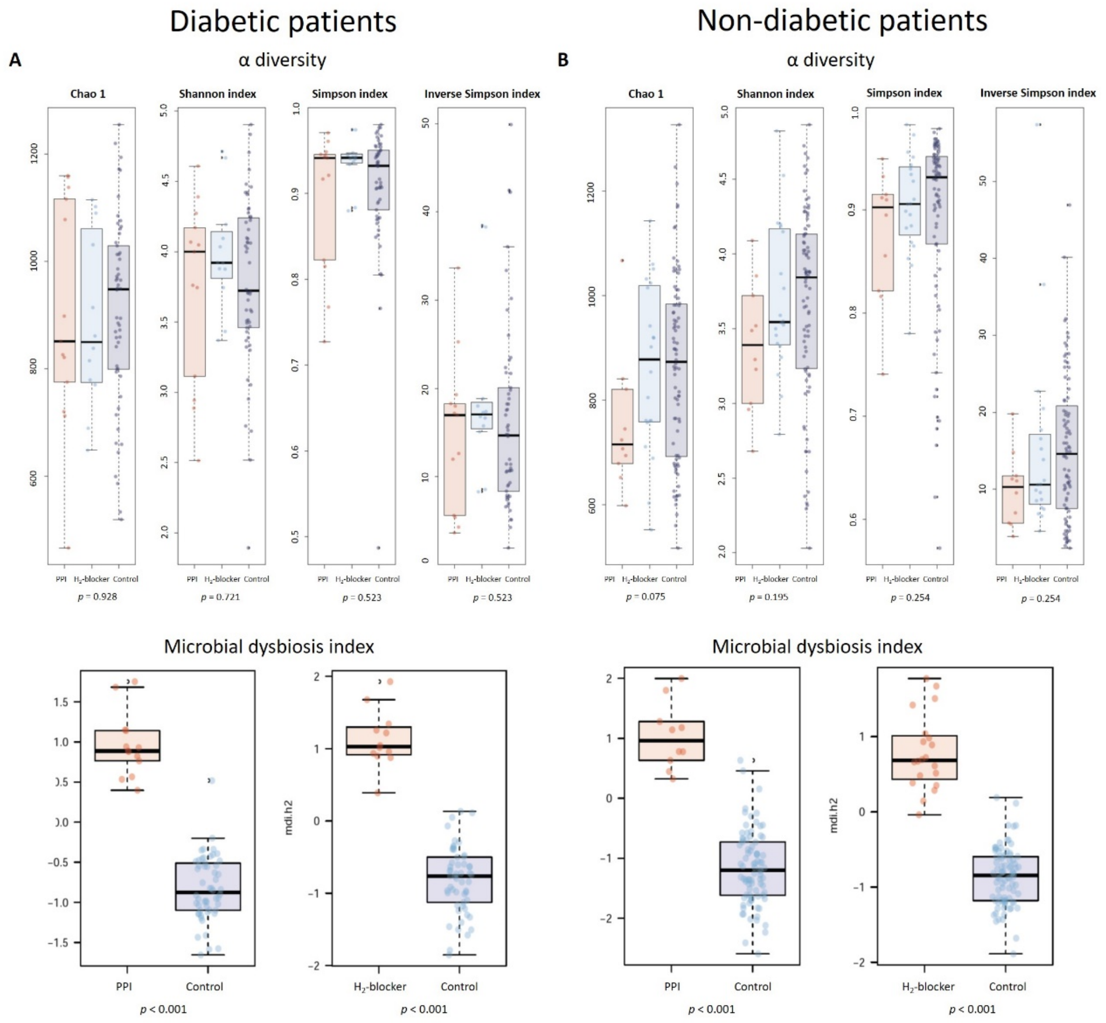

Figure S5. Core microbiome analysis in H_2-blocker users, proton pump inhibitor users, and controls; (A) SparCC correlation analysis (genus level using 100 SparCC permutations, 0.4 correlation threshold, and 0.05 P-value threshold); (B) relative abundance and sample prevalence of bacterial genus in H_2-blocker users, proton pump inhibitor users, and controls.

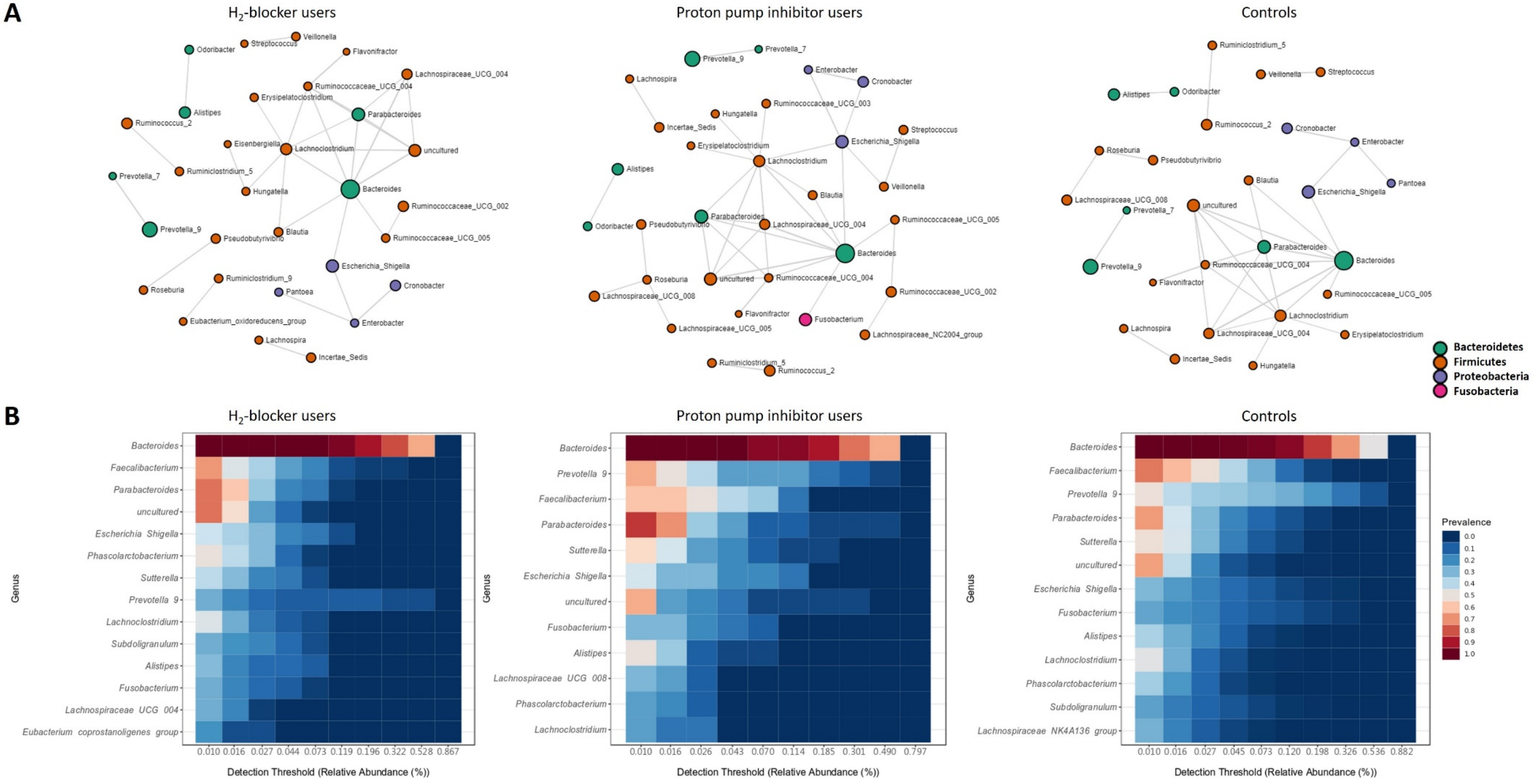

Figure S6. Hierarchical clustering heat map of bacterial genera (A) and families (B) between H_2-blocker users, proton pump inhibitor users, and controls generated by MicrobiomeAnalyst using Euclidean distance measure and Ward clustering algorithm.

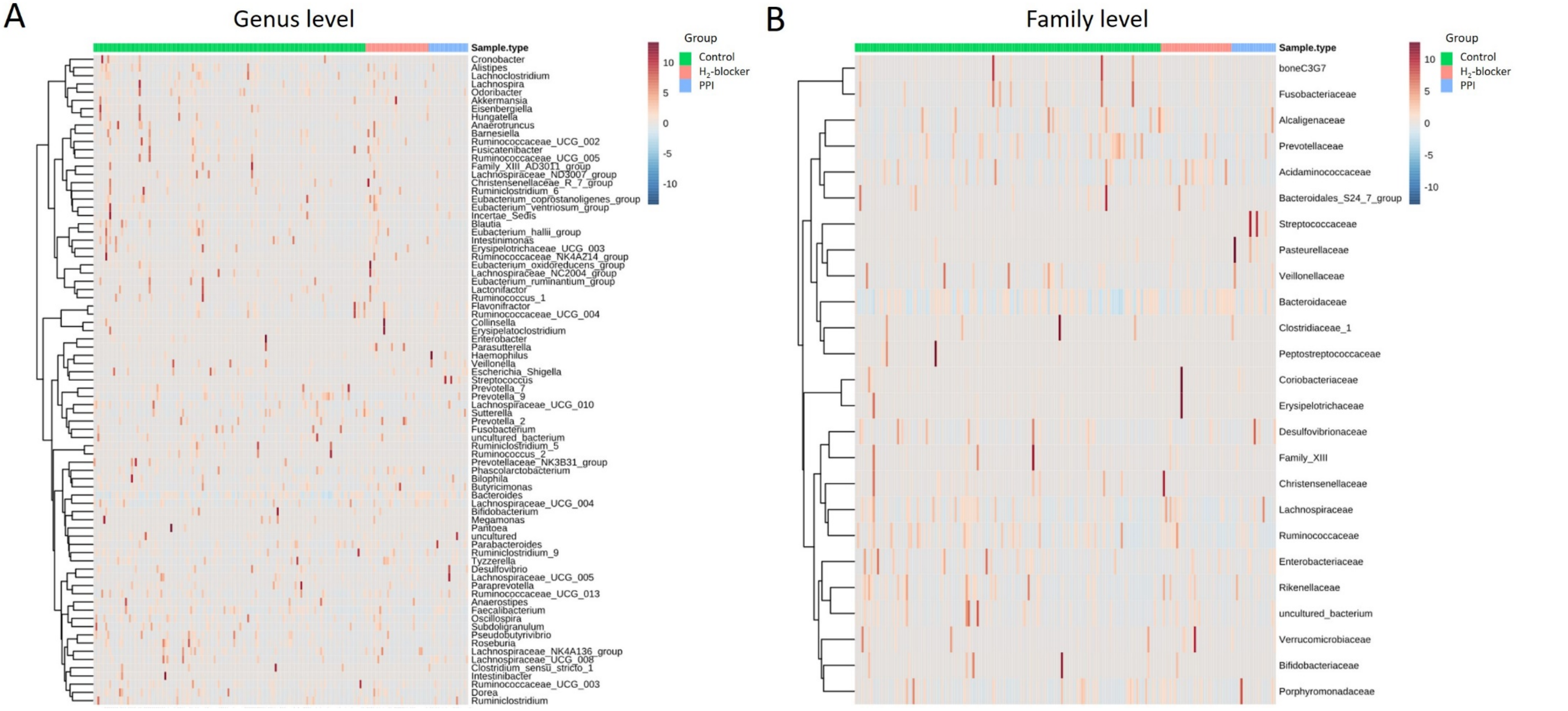

Figure S7. The abundance of the top taxa in random forest algorithm and their enriched difference between H_2-blocker users, proton pump inhibitor users, and controls: (A) species level, (B) genus level, and (C) family level.

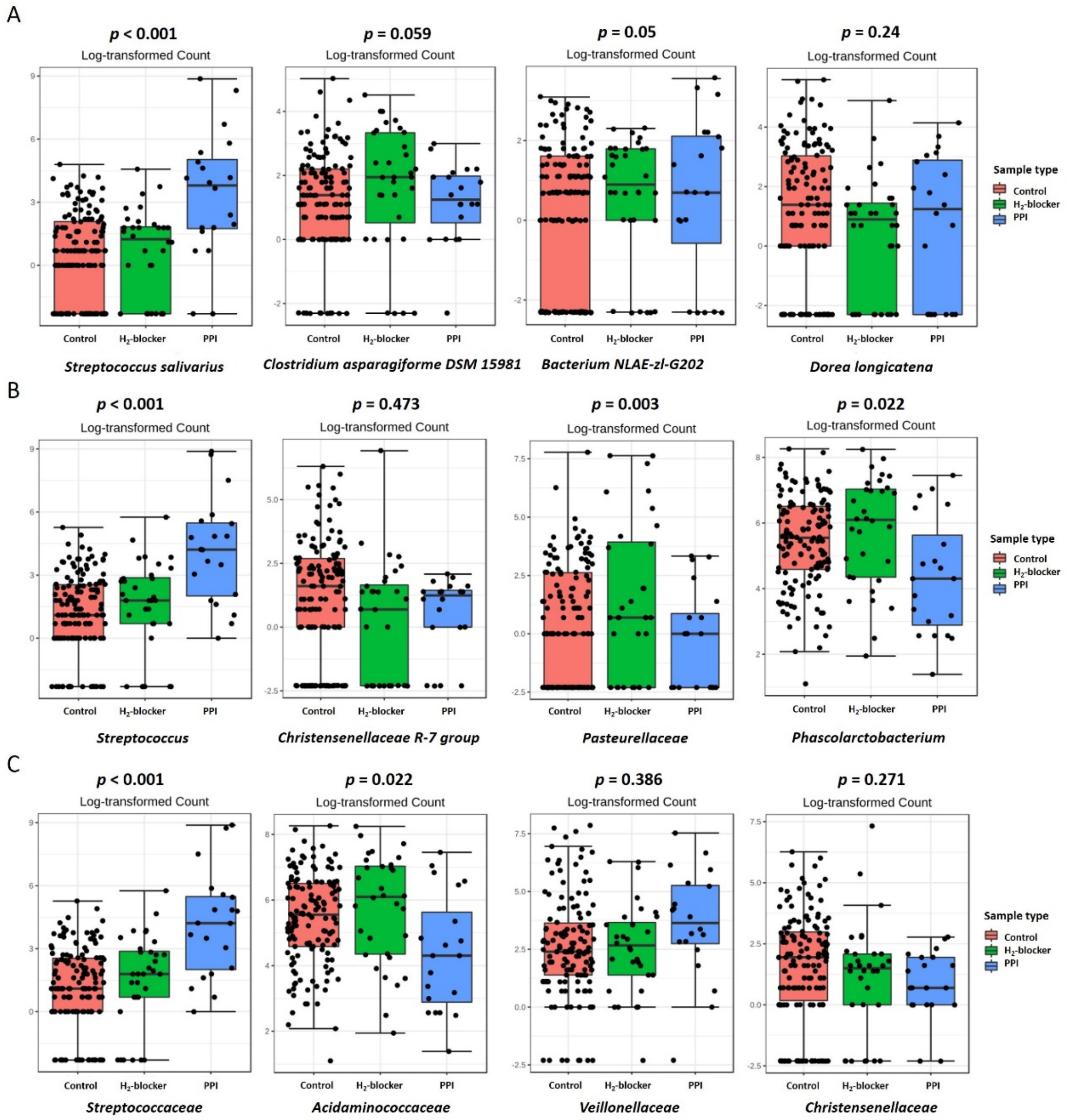

Figure S8. The relative abundance of the specific microbiome genus differentially enriched in the clinical settings after linear discriminant analysis between the H_2-blocker and proton pump inhibitor users.

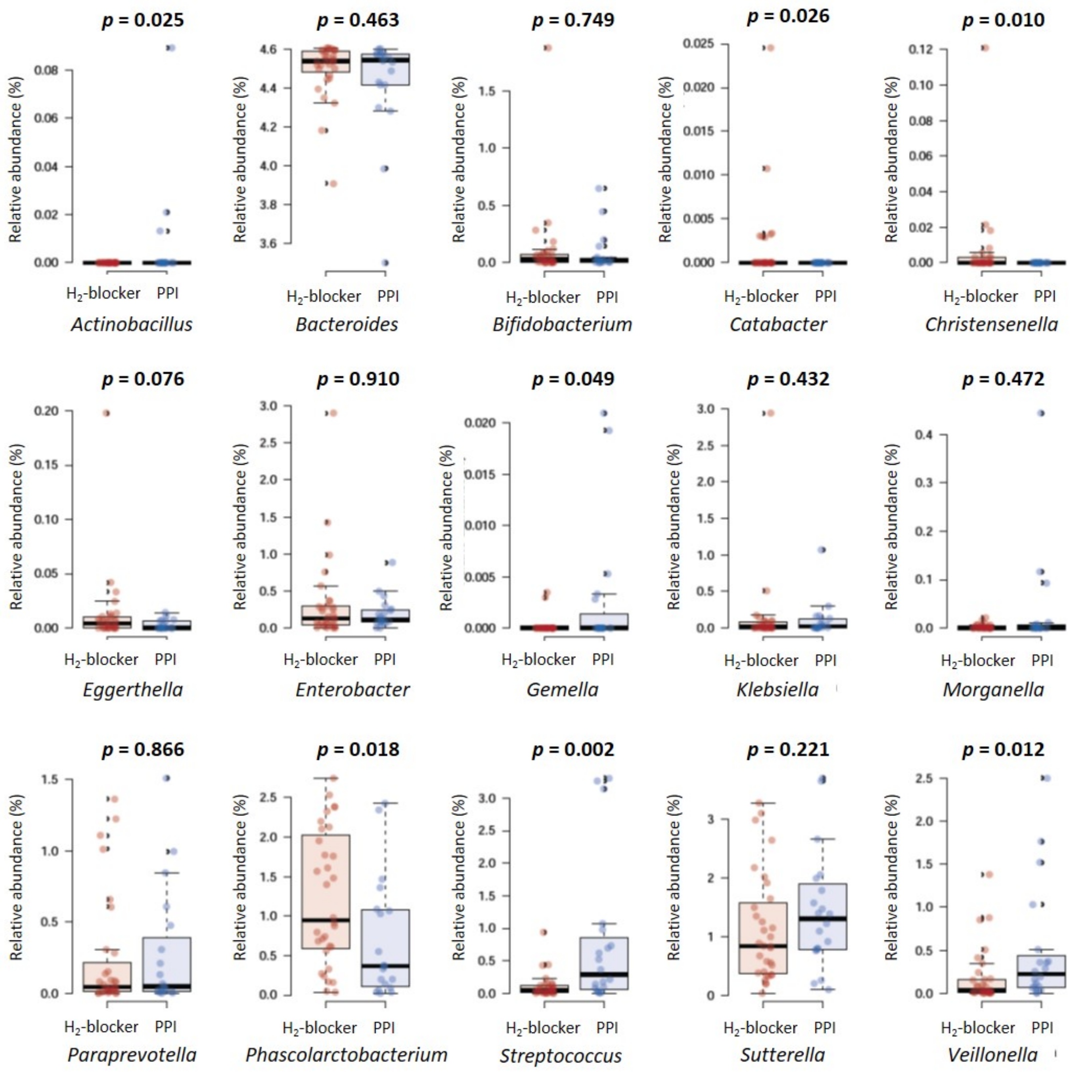

Figure S9. The relative abundance of the specific microbiome family differentially enriched in the clinical settings after linear discriminant analysis.

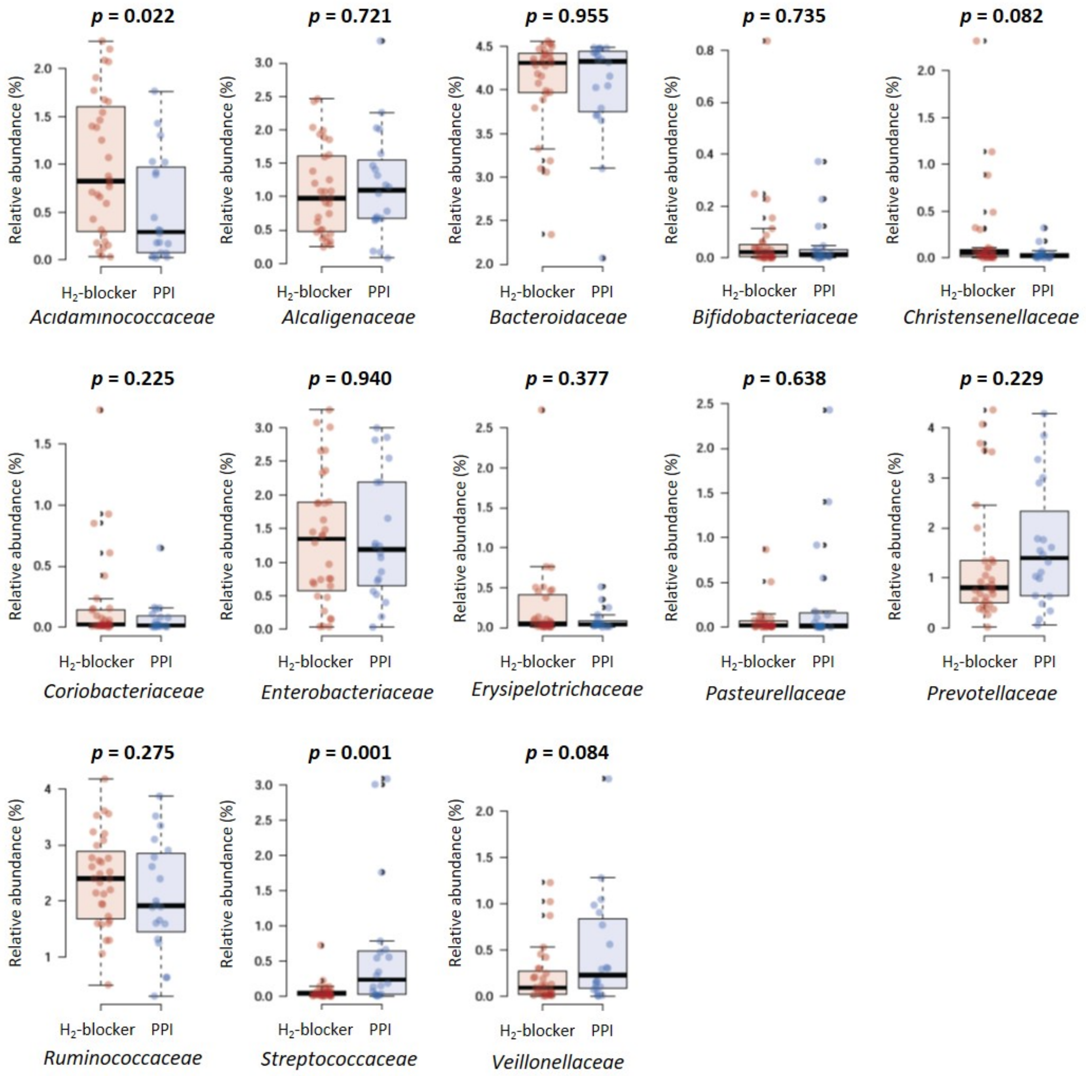

Figure S10. The relative abundance of the specific microbiome order differentially enriched in the clinical settings after linear discriminant analysis.

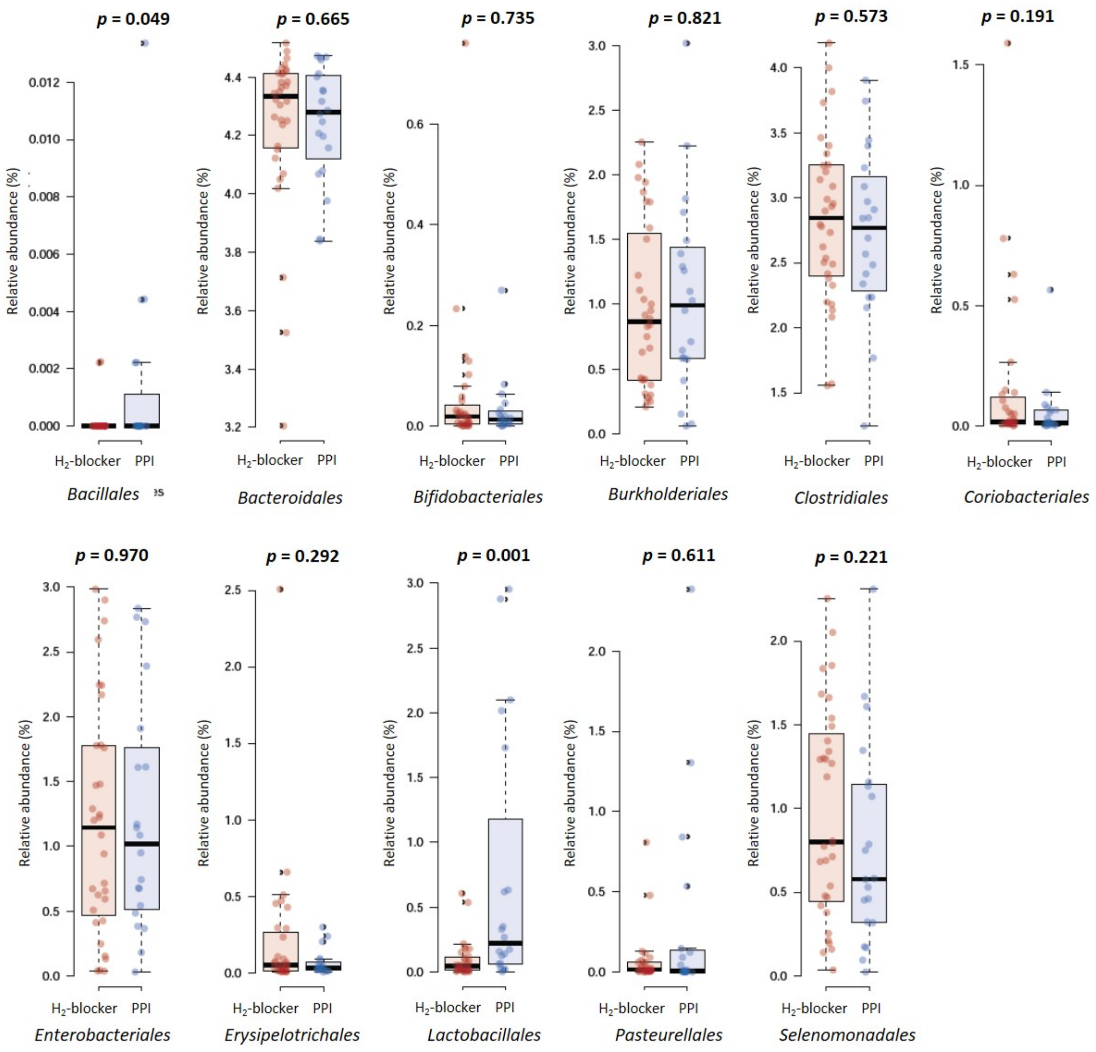

Figure S11. Determination of bacteria-specific for discriminatory across H_2-blocker and proton pump inhibitor users in hemodialysis patients. The anti-acid drugs discriminatory taxa were determined by applying random forest analysis using the (A) species-levels abundance, (B) genus-level abundance, and (C) family-level.

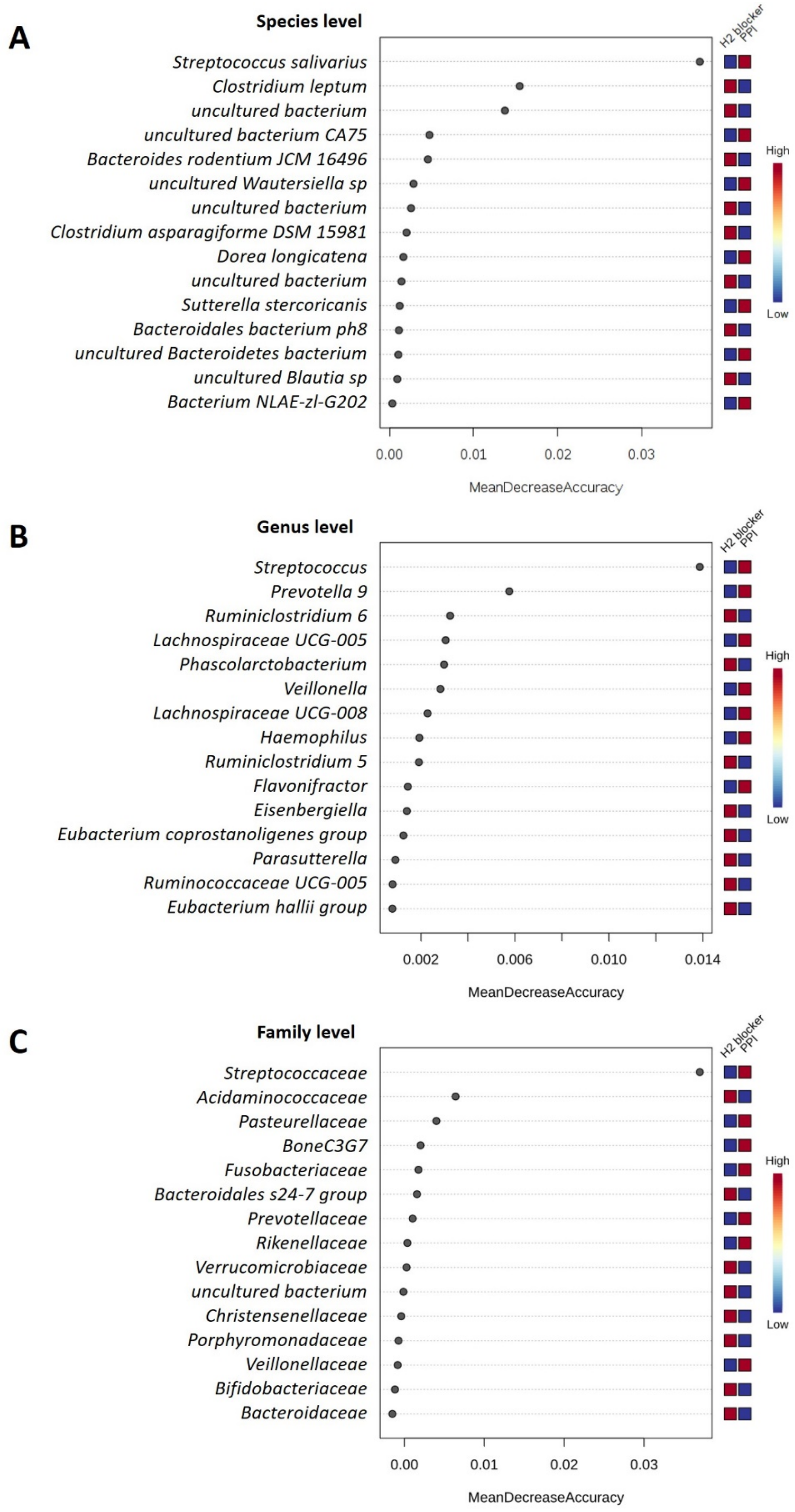

Figure S12. The abundance of the top taxa in the random forest algorithm and their enriched difference between H_2-blocker and proton pump inhibitor users: (A) species level, (B) genus level, and (C) family level.

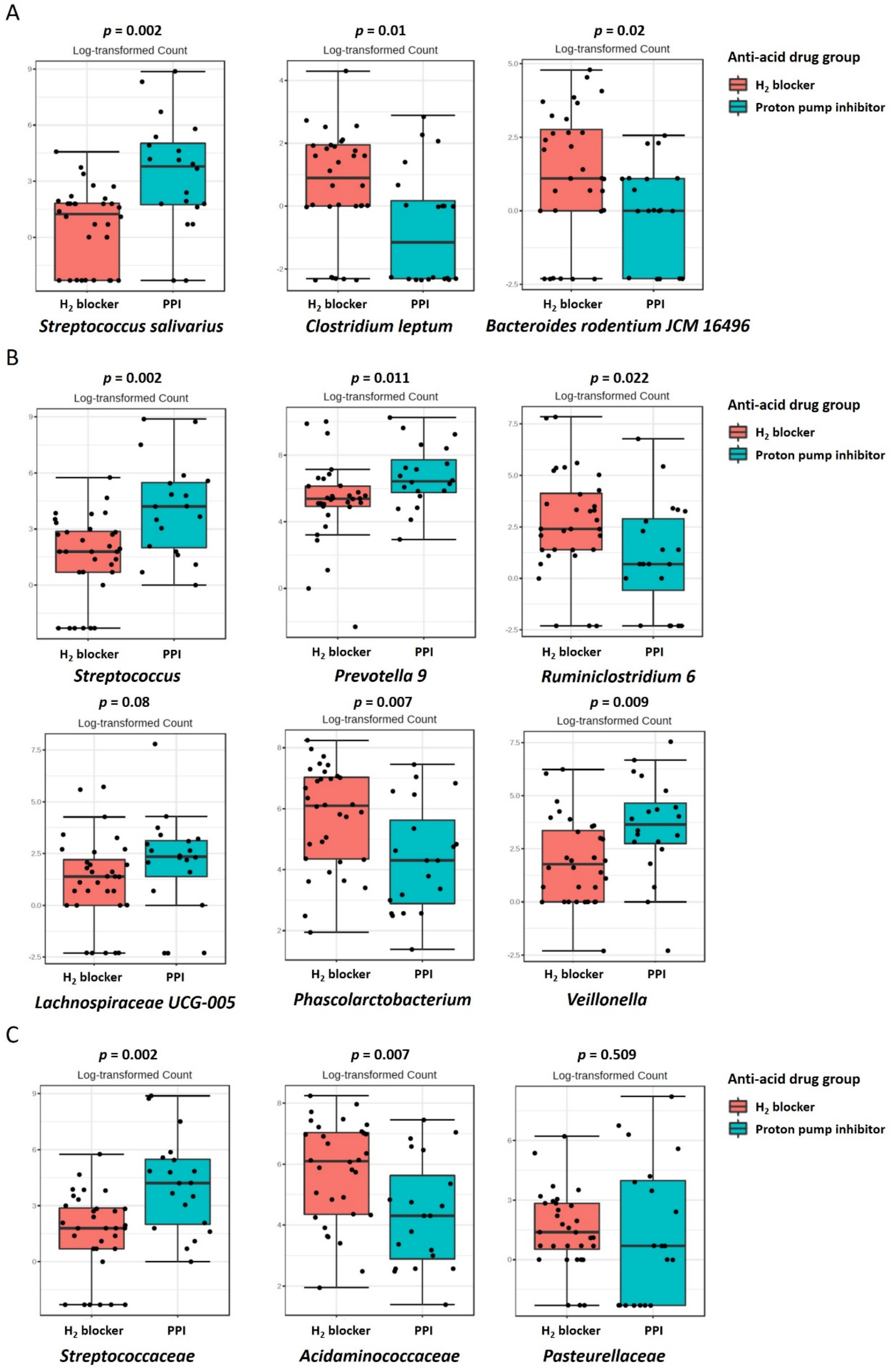

Figure S13. The β-diversity in hemodialysis patients with proton pump inhibitor users, H_2-blocker users, and controls using Human Oral Microbiome Database as the reference database (Bray–Curtis index, Jensen–Shannon divergence, and Jaccard index). Differences in β-diversity were tested by PERMANOVA.

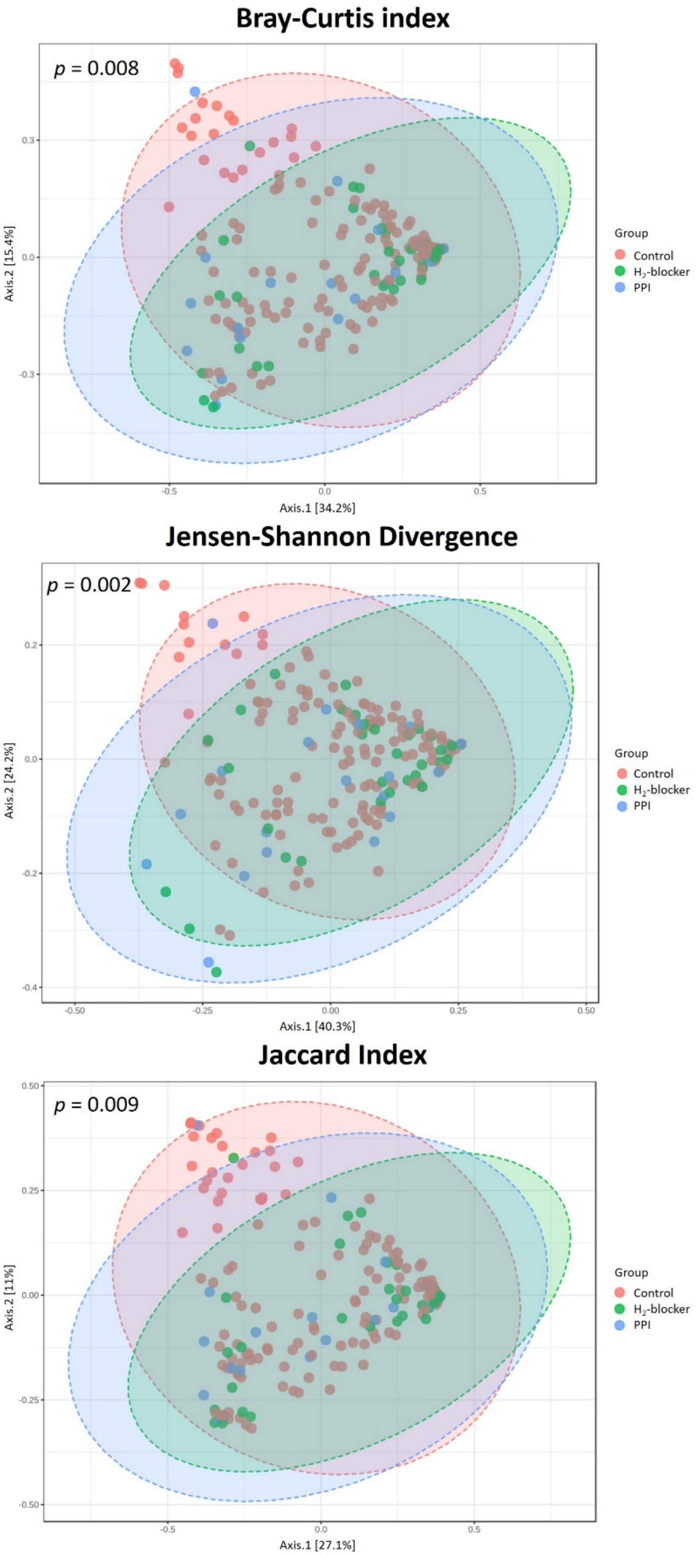

Figure S14. Heat tree visualization of taxonomic differences based on human oral microbiome database. A heat tree illustrates the taxonomic differences between H_2-blocker users, proton pump inhibitor users, and controls. The color gradient and the size of the node, edge, and label are based on the log2 ratio of median abundance: (A) proton pump inhibitor users versus controls and (B) proton pump inhibitor users versus H_2-blocker users.

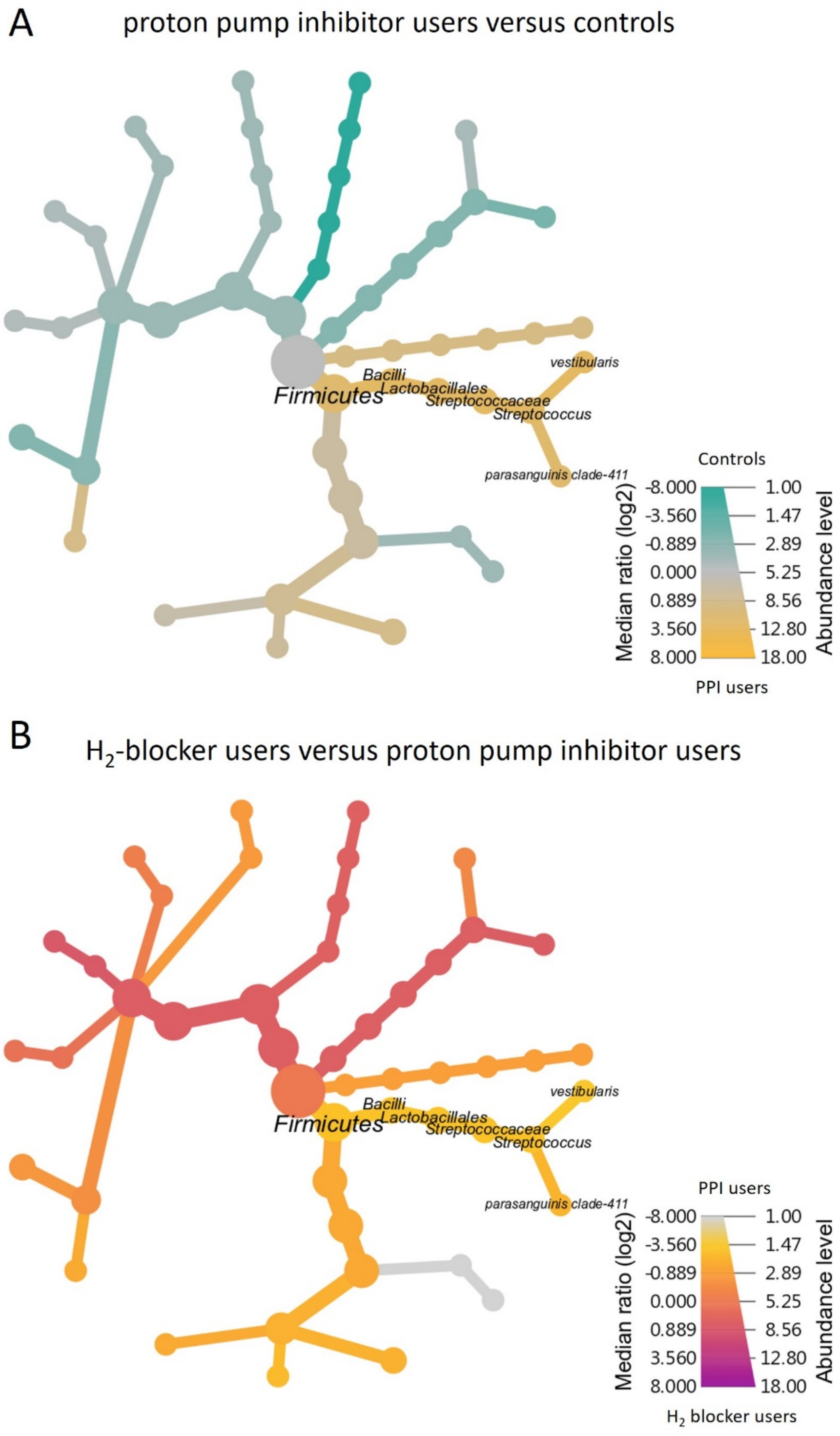

Figure S15. The abundance of the streptococcus taxa (species level, genus level, and family level) difference between H_2-blocker users, proton pump inhibitor users, and controls based on human oral microbiome database.

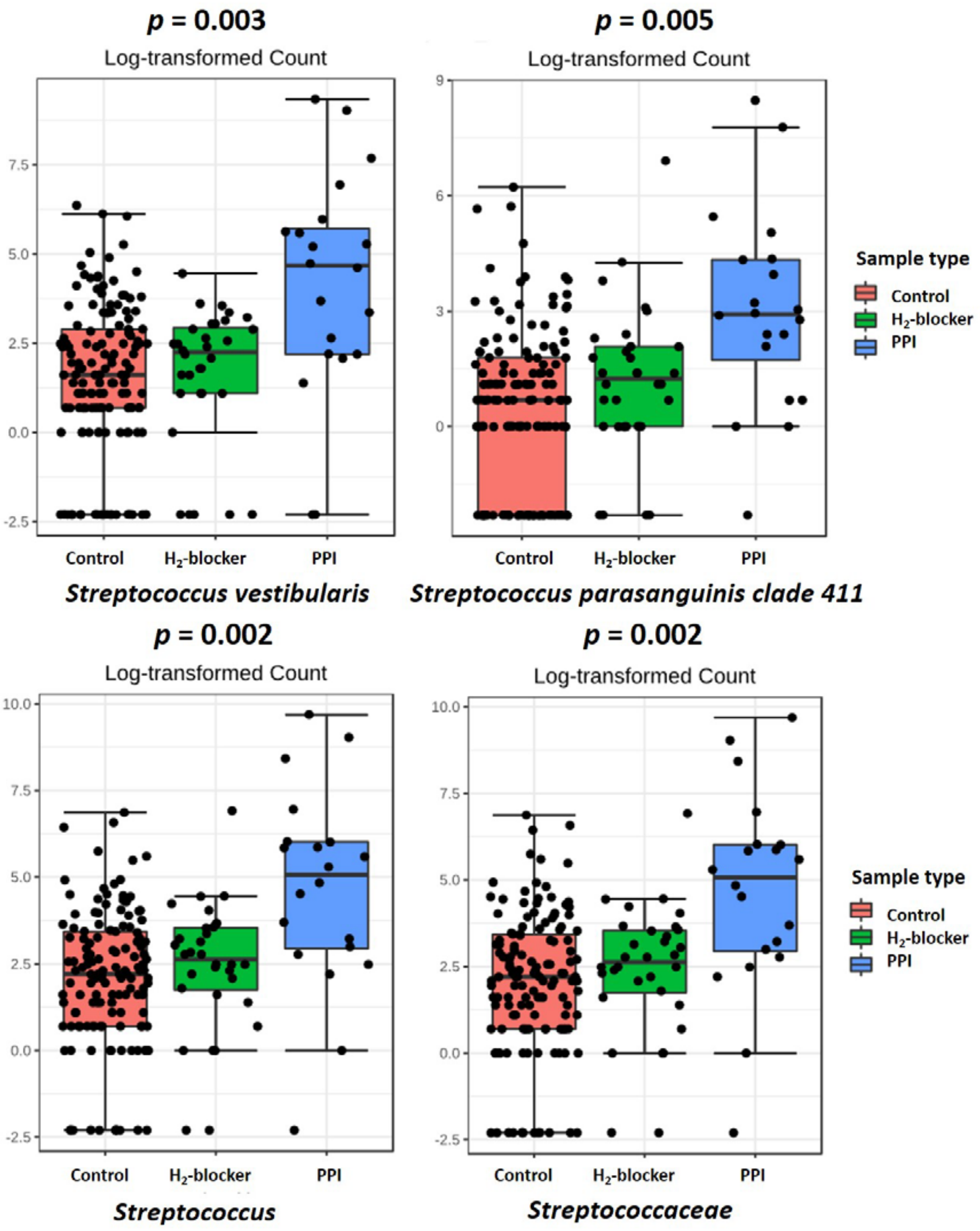

Figure S16. Selected significant functional classification of the predicted metagenome content of the microbiota of H_2-blocker users and controls by KO modules. The relative abundances of modules were compared among hemodialysis patients with and without H_2-blocker used. Significance was considered for $p < 0.05$.

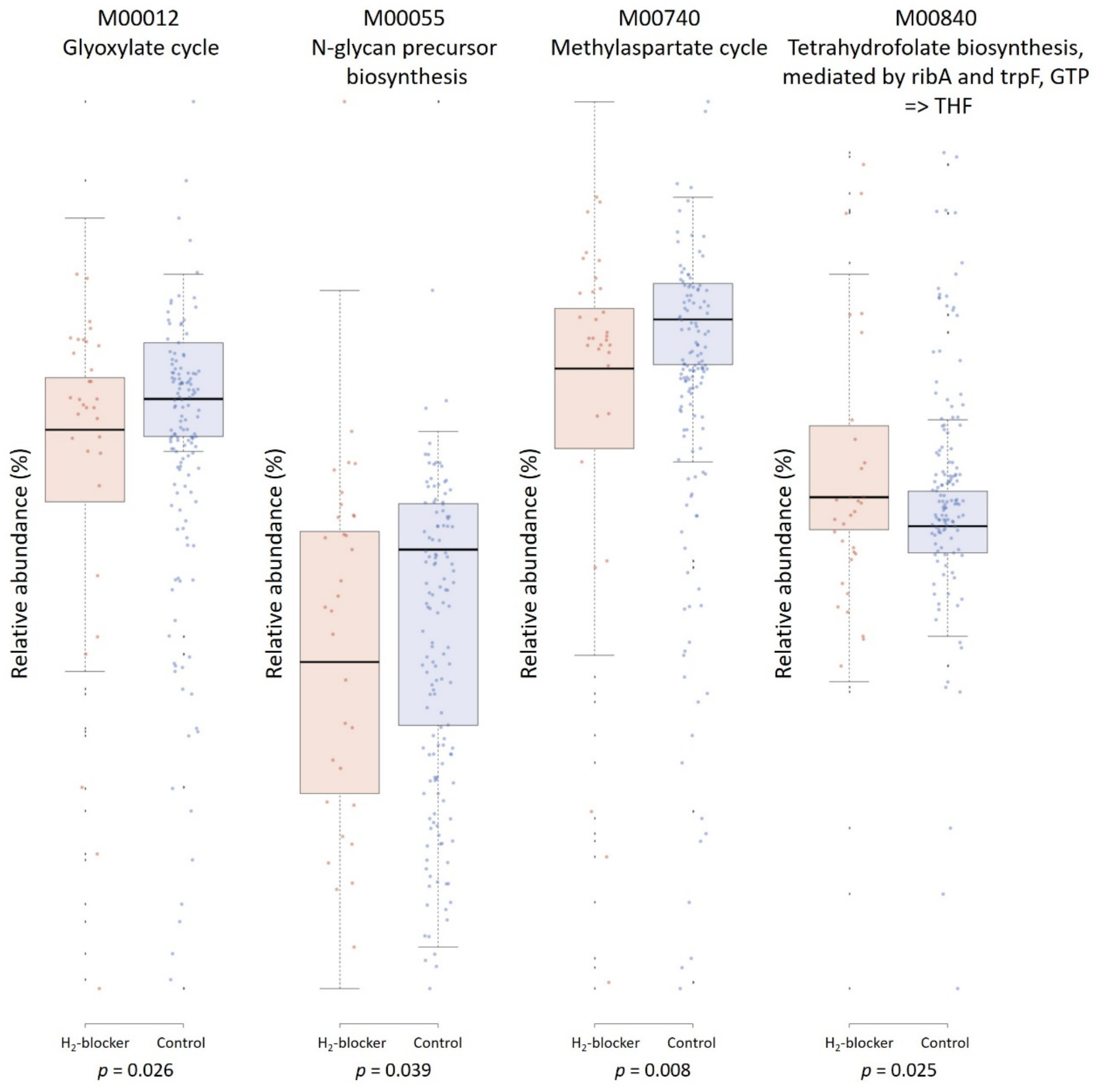

Figure S17. Selected significant functional classification of the predicted metagenome content of the microbiota of proton pump inhibitor users and controls by KO modules. The relative abundances of modules were compared among hemodialysis patients with and without proton pump inhibitors used. Significance was considered for $p < 0.05$.

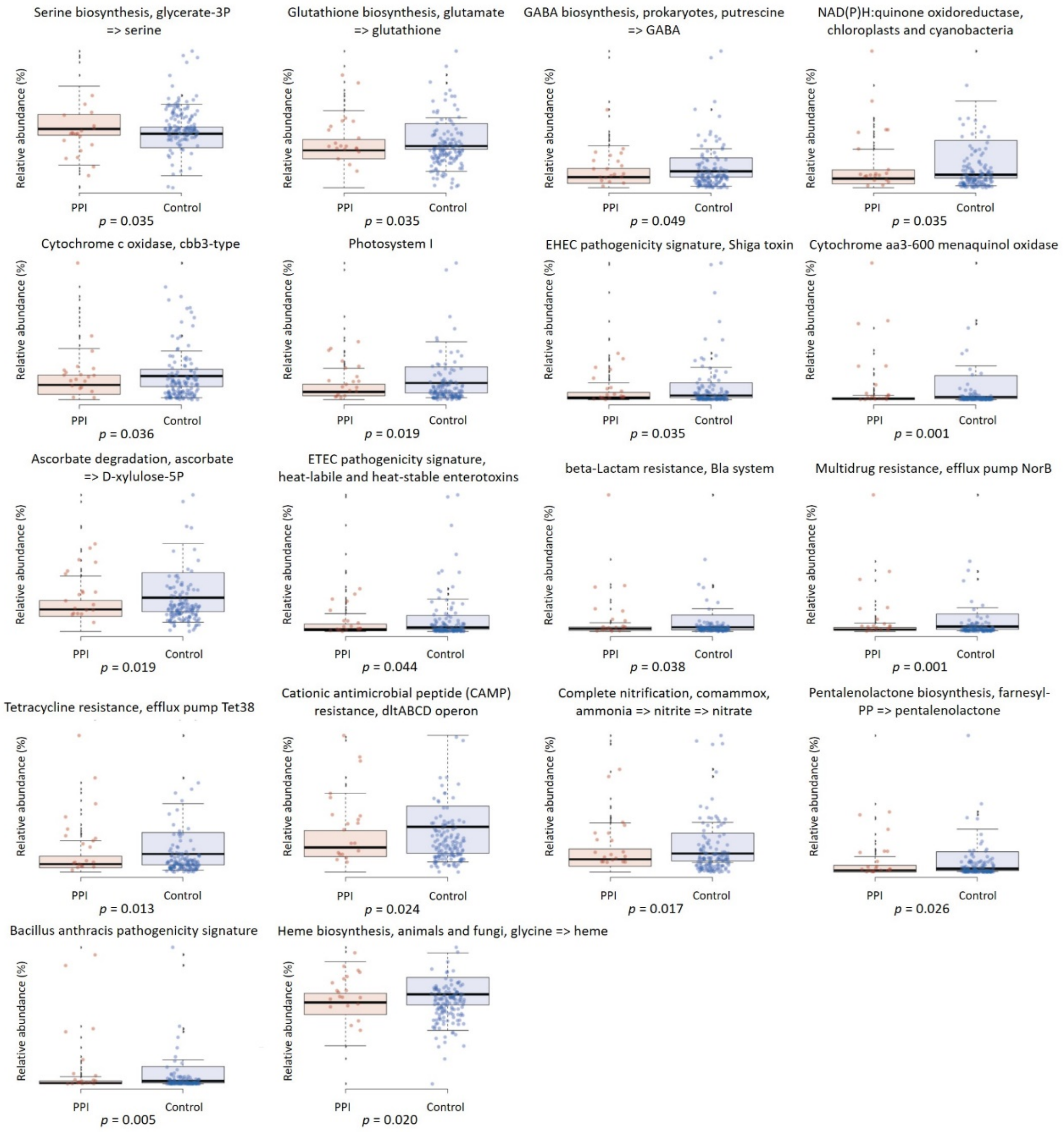

Table S1. Summary table of significant genus difference between the H2-blocker and proton pump inhibitor users in negative binomial generalized linear models (DESeq2 method) and classical univariate method.

**Genus**	**Negative binomial generalized linear models (DESeq2 method)**				**Classical univariate method**	
	log2FC	lfcSE	P	**FDR P**	P	**FDR P**
**Streptococcus**	3.45	0.68	<0.001	**<0.001**	**0.002**	0.121
**Phascolarctobacterium**	-1.851	0.551	0.001	**0.008**	**0.007**	0.197
**Veillonella**	3	0.59	<0.001	**<0.001**	**0.009**	0.197
Prevotella 9	1.343	0.587	0.022	0.112	**0.011**	0.197
**Prevotella 2**	-2.132	0.779	0.006	**0.043**	**0.021**	0.269
**Ruminiclostridium 6**	-2.697	0.705	<0.001	**0.002**	**0.022**	0.269
Eubacterium hallii group	-1.758	0.717	0.014	0.083	**0.025**	0.271
Eisenbergiella	-0.551	0.585	0.346	0.572	**0.039**	0.344
Eubacterium oxidoreducens group	-0.759	0.389	0.051	0.205	**0.041**	0.344
Parasutterella	-5.388	0.977	<0.001	**<0.001**	0.055	0.41
Lachnospiraceae UCG-008	1.89	0.43	<0.001	**<0.001**	0.069	0.46
Ruminococcaceae UCG-013	0.76	0.537	0.157	0.38	0.074	0.46
Lachnospiraceae UCG-005	1.511	0.503	0.003	0.022	0.086	0.46
Subdoligranulum	-1.855	0.623	0.003	**0.022**	0.092	0.46
Ruminococcaceae UCG-002	-0.525	0.545	0.336	0.572	0.096	0.46
Ruminococcaceae UCG-005	-1.268	0.6	0.035	0.165	0.098	0.46
Ruminiclostridium 9	-0.487	0.511	0.341	0.572	0.146	0.613
Erysipelotrichaceae UCG-003	-1.504	0.599	0.012	0.076	0.147	0.613
Blautia	-0.33	0.368	0.37	0.598	0.163	0.642
Oscillospira	-0.807	0.68	0.235	0.447	0.179	0.668
Anaerotruncus	-0.731	0.565	0.196	0.403	0.191	0.668
Uncultured bacterium	-0.686	0.348	0.049	0.205	0.213	0.668
Sutterella	0.442	0.557	0.428	0.65	0.213	0.668
Akkermansia	-0.964	0.804	0.23	0.447	0.214	0.668
Ruminococcus 1	1.403	0.847	0.098	0.284	0.235	0.678
Cronobacter	0.666	0.625	0.287	0.519	0.235	0.678
Intestinimonas	-0.928	0.675	0.169	0.38	0.247	0.685
Roseburia	0.725	0.416	0.081	0.257	0.274	0.699
Uncultured bacterium	-0.803	0.424	0.058	0.211	0.282	0.699
Hungatella	-0.869	0.487	0.074	0.246	0.301	0.699
Eubacterium coprostanoligenes group	-1.313	0.554	0.018	0.097	0.308	0.699
Fusobacterium	0.904	0.67	0.177	0.38	0.314	0.699

Incertae Sedis	0.743	0.554	0.18	0.38	0.317	0.699
Ruminococcus 2	-0.616	0.441	0.163	0.38	0.327	0.699
Prevotellaceae NK3B31 group	-1.349	1.21	0.265	0.491	0.33	0.699
Lachnospiraceae UCG-010	-0.457	0.685	0.505	0.685	0.336	0.699
Butyricimonas	-0.428	0.665	0.52	0.693	0.361	0.706
Pantoea	-0.419	0.747	0.575	0.741	0.365	0.706
Lachnospiraceae UCG-004	-0.667	0.412	0.106	0.284	0.375	0.706
Bilophila	0.463	0.566	0.413	0.641	0.377	0.706
Lachnospiraceae NC2004 group	-1.107	0.542	0.041	0.184	0.392	0.717
Prevotella 7	0.656	0.772	0.395	0.625	0.427	0.745
Bifidobacterium	0.01	0.791	0.99	0.99	0.427	0.745
Lachnoclostridium	-0.087	0.405	0.829	0.9	0.484	0.821
Tyzzerella	-0.213	0.585	0.716	0.859	0.496	0.821
Haemophilus	3.68	0.758	<0.001	**<0.001**	0.509	0.821
Desulfovibrio	0.294	0.855	0.731	0.859	0.514	0.821
Christensenellaceae R7 group	-1.073	0.567	0.058	0.211	0.539	0.835
Intestinibacter	-0.41	0.527	0.437	0.651	0.546	0.835
Clostridium sensu stricto 1	0.246	0.833	0.768	0.876	0.566	0.849
Faecalibacterium	0.238	0.513	0.643	0.801	0.608	0.859
Erysipelatoclostridium	-0.612	0.861	0.477	0.684	0.612	0.859
Ruminococcaceae UCG-003	-0.186	0.55	0.735	0.859	0.612	0.859
Fusicatenibacter	-0.103	0.531	0.846	0.905	0.625	0.859
Lachnospira	-0.067	0.469	0.886	0.924	0.638	0.859
Eubacterium ruminantium group	-3.593	1.091	0.001	**0.009**	0.642	0.859
Parabacteroides	0.256	0.349	0.463	0.677	0.675	0.888
Ruminiclostridium 5	-0.695	0.433	0.109	0.284	0.702	0.907
Eubacterium ventriosum group	-0.355	0.519	0.494	0.685	0.714	0.907
Collinsella	0.187	0.684	0.784	0.876	0.754	0.939
Ruminiclostridium	0.037	0.523	0.944	0.97	0.785	0.939
Anaerostipes	0.846	0.625	0.176	0.38	0.792	0.939
Odoribacter	-0.238	0.555	0.668	0.819	0.801	0.939
Bacteroides	-0.014	0.38	0.971	0.984	0.801	0.939
Lactonifactor	-1.452	0.776	0.061	0.212	0.816	0.942
Flavonifractor	0.508	0.526	0.334	0.572	0.851	0.948
Enterobacter	0.415	0.61	0.496	0.685	0.858	0.948
Dorea	0.261	0.437	0.55	0.72	0.859	0.948
Alistipes	-0.12	0.487	0.805	0.887	0.874	0.95
Lachnospiraceae NK4A136 group	-0.591	0.475	0.213	0.427	0.904	0.959

Paraprevotella	-1.282	0.792	0.106	0.284	0.917	0.959
Escherichia Shigella	-0.309	0.627	0.622	0.788	0.933	0.959
Pseudobutyrivibrio	-0.057	0.402	0.887	0.924	0.933	0.959
Barnesiella	-1.08	0.641	0.092	0.28	0.977	0.991
Ruminococcaceae UCG-004	0.127	0.463	0.784	0.876	0.993	0.993
Family XIII AD3011 group	-1.322	0.891	0.138	0.35	-	-
